# G1/S cell cycle regulators mediate effects of circadian dysregulation on tumor growth and provide targets for timed anticancer treatment

**DOI:** 10.1371/journal.pbio.3000228

**Published:** 2019-04-30

**Authors:** Yool Lee, Nicholas F. Lahens, Shirley Zhang, Joseph Bedont, Jeffrey M. Field, Amita Sehgal

**Affiliations:** 1 Penn Chronobiology, Howard Hughes Medical Institute, Department of Neuroscience, Perelman School of Medicine, University of Pennsylvania, Philadelphia, Pennsylvania, United States of America; 2 Institute for Translational Medicine and Therapeutics, Perelman School of Medicine, University of Pennsylvania, Philadelphia, Pennsylvania, United States of America; 3 Department of Systems Pharmacology and Translational Therapeutics, Perelman School of Medicine, University of Pennsylvania, Philadelphia, Pennsylvania, United States of America; Charité - Universitätsmedizin Berlin, GERMANY

## Abstract

Circadian disruption has multiple pathological consequences, but the underlying mechanisms are largely unknown. To address such mechanisms, we subjected transformed cultured cells to chronic circadian desynchrony (CCD), mimicking a chronic jet-lag scheme, and assayed a range of cellular functions. The results indicated a specific circadian clock–dependent increase in cell proliferation. Transcriptome analysis revealed up-regulation of G1/S phase transition genes (myelocytomatosis oncogene cellular homolog [Myc], cyclin D1/3, chromatin licensing and DNA replication factor 1 [Cdt1]), concomitant with increased phosphorylation of the retinoblastoma (RB) protein by cyclin-dependent kinase (CDK) 4/6 and increased G1-S progression. Phospho-RB (Ser807/811) was found to oscillate in a circadian fashion and exhibit phase-shifted rhythms in circadian desynchronized cells. Consistent with circadian regulation, a CDK4/6 inhibitor approved for cancer treatment reduced growth of cultured cells and mouse tumors in a time-of-day–specific manner. Our study identifies a mechanism that underlies effects of circadian disruption on tumor growth and underscores the use of treatment timed to endogenous circadian rhythms.

## Abstract 2

A study of “jet-lagged” cells reveals a specific molecular mechanism regulating cell proliferation that it impacted by circadian disruption, underscoring the importance of administering anti-cancer treatment at a specific time of day.

## Author summary

Circadian misalignment caused by altered sleep–wake cycles, shift work, or frequent jet lag increases susceptibility to several disorders, including cancer. However, the mechanisms by which circadian disruption contributes to disease are not well understood, and so we addressed this issue by investigating the molecular, cellular, and biochemical consequences of chronic circadian desynchronization. Our studies using cancer cell or tumor tissue models show that chronic circadian desynchronization induces multiple oncogenic pathways to promote cell proliferation. In particular, chronic circadian desynchronization promotes phosphorylation of the retinoblastoma (RB) protein, thereby favoring G1/S phase cell cycle progression. Consistent with these findings, the antiproliferative activity of a selective inhibitor of the enzyme that phosphorylates RB has time-of-day–specific effects on cancer cells and mouse tumors, but this time dependence is abrogated by chronic jet-lag conditions. These data suggest a circadian regulation of G1/S cell cycle progression and provide an important molecular rationale for time-of-day–specific treatment of cancer patients, also known as chronotherapy.

## Introduction

In response to day–night cycles produced by earth's 24-hour rotation around its axis, almost all living organisms have evolved circadian clocks, endogenous timekeeping systems that adapt physiology to daily changes in the environment. In mammals, the circadian timing system consists of a central light-entrained clock in the suprachiasmatic nucleus (SCN) of the brain and numerous peripheral clocks located in most body organs, all of which are typically coordinated to constrain sleep and feeding, metabolism, and immune functions to appropriate times of the day. The basic timekeeping unit is the cell in that even single neurons and fibroblasts harbor a conserved, cell-autonomous circadian clock. At the molecular level, the basic mechanism consists of transcription-translation feedback loops, with the major loop in mammals comprised of the *Period* (*Per*) and *Cryptochrome* (*Cry*) genes, which are rhythmically transcribed by circadian locomoter output cycles protein kaput (CLOCK)—brain and muscle Arnt-like protein-1(BMAL1) transcription factors and repressed by their own protein products (PER, CRY). With further fine-tuning at transcriptional, posttranscriptional, and translational levels, the intrinsic molecular oscillator integrates multiple external signals to regulate expression of clock-controlled genes, which differ dramatically from tissue to tissue despite usage of largely the same core clock genes across the organism [1].

Consistent with the adaptive physiological and cellular benefits of the circadian timing system, accumulating evidence indicates that disruption of circadian homeostasis by genetic alteration or irregular lifestyle has pathological consequences [2]. For instance, epidemiological studies show that frequent misalignment of circadian rhythms caused by lifestyle factors such as chronic sleep deprivation, jet lag, or shift work is a potential risk factor for cancer [3]. Even in experimental animal studies, genetic or environmental disruption of circadian rhythms highly increases the incidence or growth rate of various types of tumors, including lung, breast, skin, oral, and prostate cancers [4–6]. The wide variety of tumors affected suggests that circadian disruption alters basic cellular physiology in a fundamental way that increases susceptibility to diseases like cancer; however, the underlying mechanism is not understood.

Increased tumorigenesis and accelerated tumor growth are often linked to a dysregulated cell cycle [7,8]. The cell cycle consists of cell growth (G1), DNA replication (S), and cell division (G2/M) phases. Generally, control of cell proliferation occurs at the G1 phase, during which cells integrate multiple signals, ranging from growth factors to DNA damage, to determine entry into the S phase or exit to the G0 non-cycling quiescent phase [9]. Most human cancers are thought to develop from disruption of G1/S cell cycle control [10]. The G1/S phase boundary is regulated by the retinoblastoma (RB) tumor suppressor protein, which is sequentially phosphorylated by cyclin D1/cyclin dependent kinase (CDK) 4/6 in early G1 and by cyclin E/CDK2 in late G1 [11]. Links between cell cycle regulators and the circadian clock have been identified [12,13], but how the circadian clock regulates cell cycle transitions is unclear, as is the mechanism by which circadian disruption affects tumor growth.

In addition to the relevance of circadian disruption for the development of disease, circadian rhythms can be quite important for the treatment of cancer and other disorders. In recent years, some studies have investigated the idea of exploiting the circadian clock in tumors for therapy, for instance, by modulating activity of circadian clock molecules [14], enhancing intra-tumor circadian rhythms [15], and optimizing anticancer drug delivery by timing it to the host’s circadian rhythms [16,17]. Delivery timed to the appropriate time of day (chronotherapy) is expected to have better efficacy and possibly reduce the dose required, thereby limiting side effects, but the practice of chronotherapy is still in its infancy and would be facilitated by mechanistic insights into the temporal regulation of drug action.

In the present study, we sought to determine, on a cellular and molecular level, how circadian disruption affects basic cell function. Starting with cell-based circadian desynchronization experiments, we conducted extensive molecular, cellular, and biochemical analyses to reveal that chronic circadian disturbance promotes pro-proliferative signaling events, which converge to stimulate G1/S phase progression via CDK4/6-dependent RB phosphorylation. We also found that RB phosphorylation, particularly at S807/S811 sites, cycles in phase with rhythmic cyclin D1 gene expression, and it is increased and altered in fluctuation by chronic circadian perturbation. Using a mouse model, we show that circadian disruption also increases the growth of carcinogen- or melanoma-induced tumors in mice. Consistent with a role for RB-CDK4/6, we find that PD0332991, a selective CDK4/6 inhibitor, has time-of-day–specific effects on proliferation in cells and mouse tumors, but this time dependence is abrogated by a chronic jet-lag protocol. Together, our findings indicate that the circadian clock regulates G1/S phase progression via the cyclin D1-CDK4/6–RB pathway, and provide a mechanism for the application of chronotherapeutic approaches to cancer patients.

## Results

### Circadian desynchronization in a cell culture model mimics jet lag

**Fig 1:**
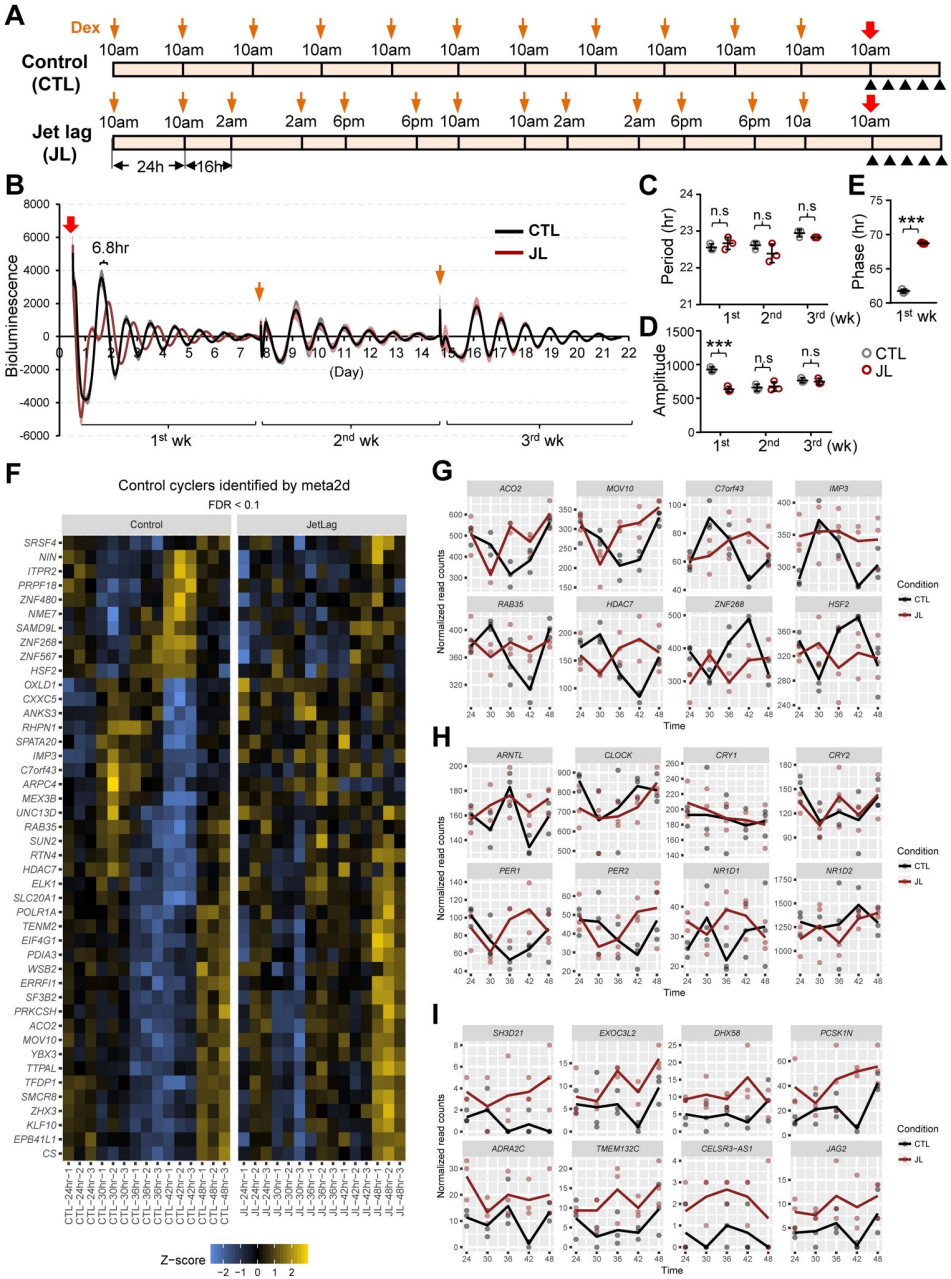
CCD alters cellular rhythms and gene expression. (A) Schematic diagram of the experimental schedule. U2OS cells stably expressing *Per2* promoter-driven destabilized luciferase (*pPer2-dLuc*) were treated with media containing 100 nM dex every 24 hours for control (Control; CTL) cells or repeated 8-hour advances of the daily cycle every 2 days to simulate jet lag (Jet lag; JL) cells for 10 days, as indicated by yellow arrows. After the CTL and JL schedule, reporter cells were subjected to real-time recording of bioluminescence activity, as indicated by red arrows. Black arrowheads indicate the sampling time, every 6 hours, for analyses of circadian gene expression 24 hours after the final dex treatment. (B) Bioluminescence recordings of dex-synchronized cells subjected to a CTL or JL schedule described in (A) (left). The data are plotted with results from three cultured dishes representing each condition, CTL (black) and JL (brown), followed by a 24-hour moving average subtraction. Shading indicates the standard deviation for each point. The red arrow indicates the start of the bioluminescence measurement of CTL and JL cells following the final dex treatment. The yellow arrows indicate the dex stimulations every week (1 week, 2 weeks, 3 weeks). (C, D, E) Period (C), amplitude (D), and phase (E) analysis of circadian bioluminescence data of CTL (grey circle) and JL (brown circle) cells for the indicated weeks (1 week, 2 weeks, 3 weeks). ***p < 0.0001; two-way ANOVA and Bonferroni multiple comparisons test (C and D). **p < 0.001; two-tailed Student *t* test (E) (n.s., *p* > 0.05). Representative data from four independent experiments are shown as mean ± SD. *n* = 3. (F) Chronic desynchronization alters circadian cycles of gene expression. The heat map displays expression patterns of cycling genes identified by MetaCycle (q < 0.1) from the RNA-seq time course (depicted by black arrows in panel A). Color is scaled by calculating z-scores from normalized RNA-seq read counts within each row. Underlying data are provided in S1 Data. (G, H, I) RNA-seq expression traces from CTL (black) and JL (brown) samples for genes exhibiting altered expression rhythms due to CCD (G), core clock genes affected by CCD (H), and (I) genes with the highest fold-change differences between all JL samples and all CTL cells (see S1, S2 and S3 Tables). Underlying data are provided in S2 Data. Dots indicate expression levels for individual replicates, while lines connect means of the three replicates at each time point. Underlying data for this figure can be found in S3 Data. CCD, chronic circadian desynchrony; CTL, control; dex, dexamethasone; FDR, false discovery rate; n.s., not significant; JL, jet lag; *Per2*, *Period2*; RNA-Seq, RNA sequencing; U2OS, human U2 osteosarcoma.

Experimental disruptions of the circadian clock, such as chronic jet lag in mice and rats, have detrimental effects on health, causing aberrant behavioral and hormonal rhythms, cognitive deficits, weight gain, diabetes, apoptosis, and accelerated tumor development and growth [18]. However, molecular, biochemical, or cellular mechanisms underlying these effects of circadian disturbance remain ambiguous. In order to determine consequences of circadian disruption on the most basic level, we sought to develop a cell-based, chronic circadian desynchrony (CCD) assay mimicking the experimental jet-lag protocol used in mouse models [4,19]. To test the feasibility of an in vitro CCD approach, we chose a commonly used circadian model [20], human U2 osteosarcoma (U2OS) cells expressing a *Period2* (*Per2*) promoter-driven destabilized luciferase reporter (*pPer2-dLuc*). This reporter cell line enables real-time measurement of cellular rhythms after synchronization with dexamethasone (dex), a synthetic glucocorticoid (GC) and potent clock synchronizer widely used in cell culture models ranging from fibroblasts to astrocytes [21–23]. First, to assess the effect of CCD on cellular rhythmicity, we exposed control cells to 10 days of a regular change of dex (100 nM)-containing media at 24-hour intervals (Control; CTL), while experimental cells received serial 8-hour advances of the dex treatment cycle every 2 days (Jet lag; JL) (Fig 1A).

After the CCD schedule, dex synchronization was done on a weekly basis to prevent decoupling of circadian phases across cells, and real-time activity of the *Per2* promoter was recorded in both CTL and JL cells (Fig 1B). Analysis of bioluminescence rhythms during the first week (1^st^wk) post CCD revealed significantly dampened rhythms in JL cells, with lower amplitude and, notably, 6.8-hour delayed onset of rhythms (acro-phase) (Fig 1C, 1D and 1E). However, rhythms in JL cells were not different from those of CTL cells in subsequent weeks (2^nd^wk, 3^rd^wk), throughout which all cells showed nearly identical rhythmic cycles in luminescence (Fig 1B, 1C and 1D). These effects are similar to those that occur with travel across different time zones, when rhythms are affected in the first week following travel but subsequently adapt to the new, stable environment [24]. A phase delay (8.2 hours) and reduced amplitude of JL cell rhythms were also observed in the first week after a short-term circadian desynchronization schedule of only 6 days (S1 Fig).

The observations above concur with a previous mouse study showing that temporal patterns of circadian clock genes (*Per1*, *Per2*, *Cry1*) in chronically jet-lagged mice exhibit lower amplitude as well as phase delays of 5.5–9.0 hours and 7.0–11.2 hours in the SCN and liver, respectively [19]. Moreover, in a recent human study, a similar set of circadian genes (*PER1*, *PER2*) as well as hormones (plasma cortisol, melatonin) showed between a 7- and 9-hour phase delay with decreased amplitude in human blood samples after a night shift protocol [25]. Taking this evidence together, we conclude that our cultured cell-based CCD strategy closely mimics a physiological jet-lag model.

### Circadian desynchrony affects cell physiology to selectively increase proliferation

To determine the effects of CCD on cellular function and physiology, we conducted a wide range of assays that tested for oxidative stress/senescence (H_2_O_2_, glutathione [GSH]/glutathione disulfide [GSSG]), metabolism (NADP/NADPH), proteolysis, and apoptosis, but we did not find any significant differences between CTL and JL cells (S2 Fig). To take a more unbiased approach, we investigated genome-wide changes of rhythmic gene expression following the CCD schedule. To this end, we performed RNA sequencing (RNA-Seq) analysis of CTL and JL cell samples over a 24-hour cycle collected every six hours (Fig 1A; black arrowheads). MetaCycle [26] analysis revealed significant circadian cycles of expression (q < 0.1) for 44 transcripts in CTL cells (Fig 1F and 1G, and S1 Table). Interestingly, in JL cells, the transcriptional rhythms of these cyclers were severely compromised or lost (Fig 1F and 1G, and S2 Table). The expression of several known circadian genes also showed dampened or phase-altered oscillations in JL cells (Fig 1H). Differential expression (DE) analysis comparing all CTL samples to all JL samples revealed many genes significantly up-regulated at all circadian times in JL cells (S3 Table). Interestingly, many genes up-regulated in JL cells function in disease pathways implicated in neurodegeneration and cancer: SRC homology domain-containing protein 21 (SH3D21) (ataxia-telangiectasia and colorectal cancer [27]), exocyst complex component 3-like protein 2 (EXOC3L2) (Alzheimer disease [28]), DEXH (Asp-Glu-X-His) box polypeptide 58 (DHX58) (mammary tumor [29]), proprotein convertase subtilisin/kexin type 1 inhibitor (PCSK1N) (skin carcinogenesis, Alzheimer disease and parkinsonism-dementia [30]), and jagged 2 (JAG2) (myeloma [31]) (Fig 1I and S3 Table). These observations indicate that in vitro chronic circadian disturbance significantly perturbs cellular rhythmicity as well as disease-relevant pathways.

**Fig 2:**
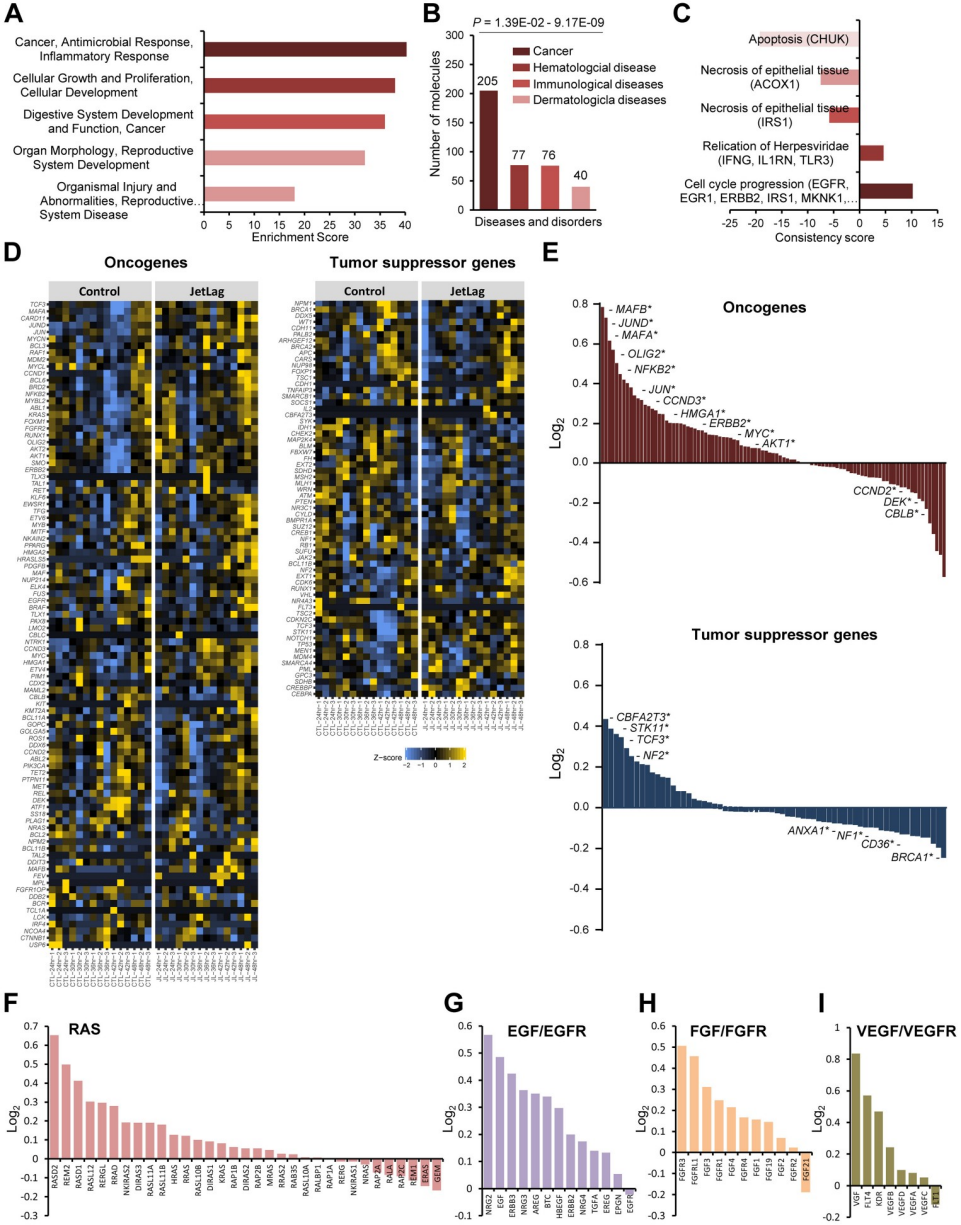
Chronic jet lag promotes a pro-proliferative cellular environment. (A, B, C) Biological functions and top gene networks (A), disease pathways (B), and effector networks (C) implicated by genes expressed differentially between control (CTL) and jet lag (JL) cells. Ingenuity Pathway Analysis (IPA) was used for this determination. (D) Heat map displays expression patterns of well-characterized oncogenes and tumor suppressor genes in CTL and JL cells, from RNA sequencing data. (E) Log_2_ fold-change values for oncogenes and tumor suppressor genes shown in (D). The asterisk indicates statistically significant differences (*p* < 0.05). Underlying data are provided in S4 Table. (F-I) Log2 fold-change values for gene expression of key ligands and receptors of (F) RAS, (G) EGF/EGFR, (H) FGF/FGFR, (I) VEGF/VEGFR signaling pathways. Fold-change values calculated using RNA-sequence data from CTL and JL cells (See S5, S6, S7 and S8 Tables). Underlying data for this figure can be found in S3 Data. CTL, control, EGF, epidermal growth factor; EGFR, epidermal growth factor receptor; FGF, fibroblast growth factor; FGFR, fibroblast growth factor receptor; IPA, Ingenuity Pathway Analysis; JL, jet lag; RAS, retinoic acid syndrome; VEGF, vascular endothelial growth factor; VEGFR, vascular endothelial growth factor receptor.

Pathway analysis of DE genes (CTL versus JL samples; q < 0.4) found enrichment of gene networks involved in cellular growth, proliferation, and cancer (Fig 2A, 2B and 2C).

Prompted by this, we compared gene expression patterns between CTL and JL samples for well-characterized oncogenes and tumor suppressor genes. We found that several oncogenes (56/95) were up-regulated in JL cells, with significant induction noted for 11 genes (e.g., musculoaponeurotic fibrosarcoma oncogene B [MAFB], Jun proto-oncogene related gene d [JUND], musculoaponeurotic fibrosarcoma oncogene B [MAFA], oligodendrocyte transcription factor 2 [OLIG2], nuclear factor kappa B subunit 2 [NFKB2], jun oncogene [JUN], cyclin D3 [CCND3], high mobility group AT-hook 1 [HMGA1], erbb2 receptor tyrosine kinase 2 [ERBB2], myelocytomatosis oncogene cellular homolog [MYC], alpha serine/threonine kinase 1 [AKT1]; *p* < 0.05). On the other hand, tumor suppressor genes (58/66) were largely unaffected or significantly down-regulated (e.g., annexin A1 [ANXA1], neurofibromin 1 [NF1], cluster of differentiation 36 [CD36], breast cancer type 1 susceptibility protein [BRCA1]; *p* < 0.05) (Fig 2D and S4 Table). This gene expression pattern is more clearly visible when plotting the log_2_ fold-change values for oncogenes and tumor suppressor genes (Fig 2E). Overall expression of oncogenic retinoic acid syndrome (RAS) signaling factors as well as receptors and ligands of several growth factor signaling pathways (epidermal growth factor [EGF]/fibroblast growth factor [FGF]/vascular endothelial growth factor [VEGF]), which contribute to cancer progression, was also induced in response to CCD (Fig 2F–2I; S5, S6, S7 and S8 Tables). These data indicate that CCD reprograms the transcriptional landscape of multiple tumor progression or suppression pathways to facilitate cell proliferation and survival.

**Fig 3:**
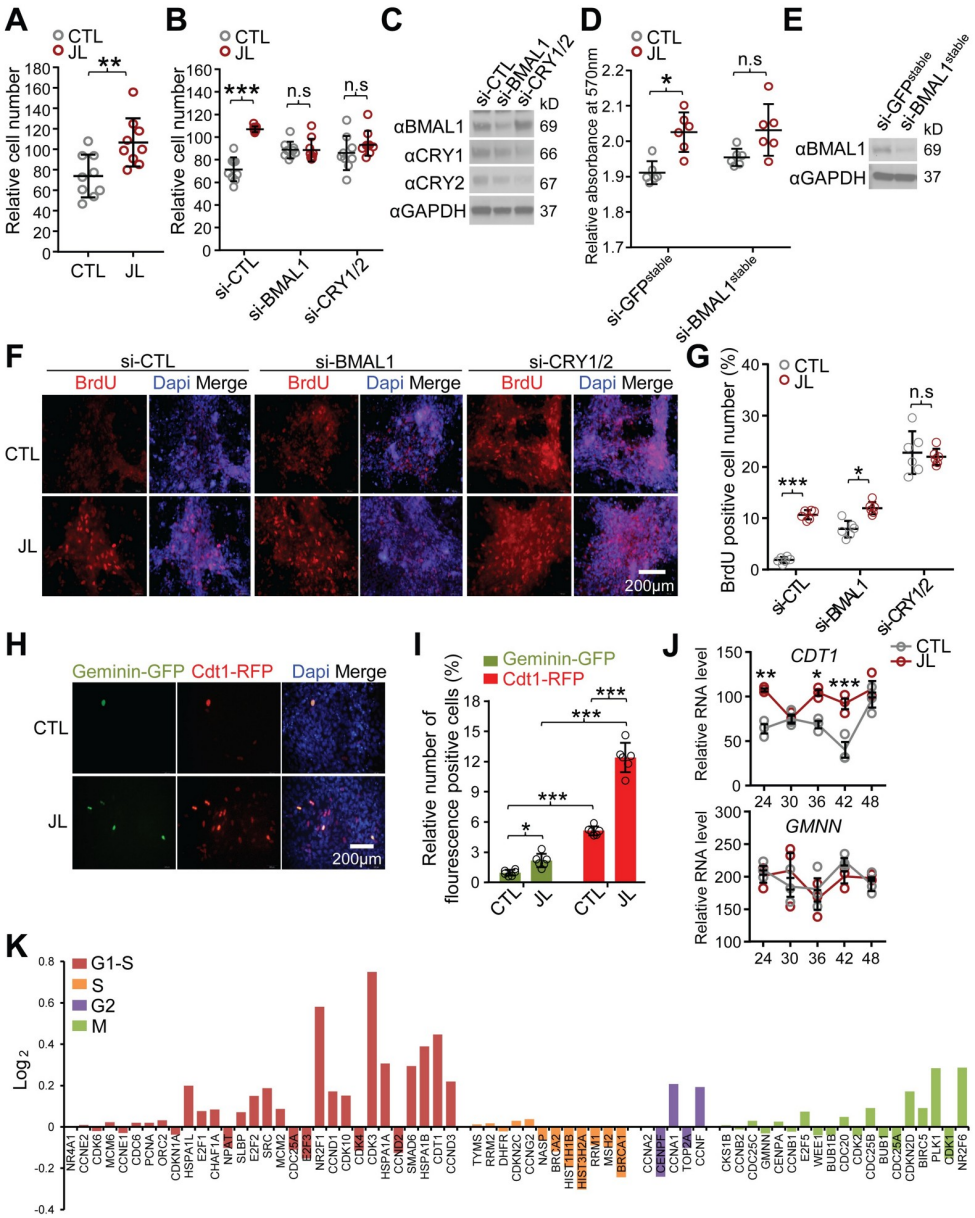
CCD enhances cell proliferation. (A) Twenty-four hours after the final dex stimulation, as per the experimental schedule depicted in Fig 1A, the control (CTL: grey circle) and jet lag (JL: brown circle) cells were harvested and subjected to a cell viability assay with 0.4% trypan blue to determine cell numbers. **p < 0.001, two-tailed Student *t* test. Data are presented as mean ± SD; *n* = 9 samples from three independent experiments. (B) Knockdown of core clock regulators abrogates the effect of chronic jet lag on cell proliferation. Forty-eight hours post-transfection of control siRNA (si-CTL) or siRNAs targeting BMAL1 (si-BMAL1) or both CRY1 and CRY2 (si-CRY1/2), the CTL (grey circle) and JL (brown circle) cells were subjected to the same experimental procedures as described in (A). ***p < 0.001; two-way ANOVA and Tukey multiple comparisons test. Data are presented as mean ± SD; *n* = 9 samples from three independent experiments. (C) Western blot analysis of knockdown efficiency of si-BMAL1 or si-CRY1/2 used in (B). Anti-BMAL1, anti-CRY1, and anti-CRY2 antibodies were used for detecting endogenous BMAL1, CRY1, and CRY2. Anti-GAPDH (αGAPDH) was used for loading control. (D) Evaluation of cell proliferation by MTT assay in U2OS cells stably expressing siRNA against GFP (si-GFP^stable^) or BMAL1 (si-BMAL1^stable^) following the CTL: grey circle) and JL (brown circle) dex synchronization schedules. *p < 0.05; two-way ANOVA with Tukey multiple comparisons test. Data are shown with the means ± SEM; *n* = 6 in all groups. (E) Western blot analysis of BMAL1 knockdown efficiency in the BMAL1 (si-BMAL1^stable^) or GFP siRNA (si-GFP^stable^) transgenic cells using anti-BMAL1 antibody. Anti-GAPDH (αGAPDH) was used for loading control. (F) BrdU incorporation assay to assess cell proliferation in response to chronic desynchronization in control siRNA (si-CTL) versus BMAL1 or CRY1/2 siRNA-transfected U2OS cell cultures. Representative images show BrdU immunolocalization (red) in cell nuclei counterstained with Dapi (blue) 24 hours post control and jet-lag dex synchronization schedules, as depicted in Fig 1A. Scale bar, 200 μm. (G) Statistical analysis quantifying the fraction of BrdU-positive proliferating cells in (F). Proportion of anti-BrdU-positive cells from the total number of Dapi-stained nuclei (>200) in each of the si-CTL-, si-BMAL1–, or si-CRY1/2–transfected cultures after the CTL (grey bar) or JL (dark brown bar) schedule was averaged from six optical fields scanned with a 20× objective. *p < 0.05, ***p < 0.0001; two-way ANOVA with Tukey multiple comparisons test. Data are shown with the means ± SD; *n* = 6 per group. n.s., *p* > 0.05. (H) At the final dex-containing media change during the chronic desynchronization schedule, the CTL and JL cells were transduced with baculovirus-expressing FUCCI cell cycle sensors (Cdt1-RFP for G1/S, Geminin-GFP for G2/M) for 48 hours and fixed for microscopic analysis. Representative images were captured by fluorescence imaging microscopy using specific filter sets for FITC (grey green; Geminin-GFP), TRITC (red; Cdt-RFP), and DAPI (blue; nuclei). Scale bar, 200 μm. (I) Quantification of the fraction of FUCCI cell cycle indicator–positive cells shown in (H). Proportion of Geminin-GFP (green bar)–or Cdt1-RFP (red bar)–positive cells from the total number of Dapi-stained nuclei (>200) in the CTL or JL cells were averaged from six optical fields scanned with a 20× objective. *p < 0.05, ***p < 0.0001; two-way ANOVA with Tukey multiple comparisons test. Data are shown with the means ± SEM; *n* = 6 per group. (J) Comparison of *CDT1* and *GMNN* mRNA expression profiles from RNA sequencing data with CTL (grey circle) and JL (brown circle) cells collected every 6 hours, as indicated, for 24 hours following the chronic desynchronization schedule depicted in Fig 1A. *p < 0.05, **p < 0.005, **p < 0.001; two-way ANOVA with Tukey multiple comparisons test. Data are shown with the means ± SEM; *n* = 3 in all time points. (K) Log_2_ fold-change values for cell cycle–specific genes, colored by associated phases (G1/S, S, G2, M). Fold-change values were calculated using RNA-Seq data of CTL and JL cells presented in S3A Fig. See S9 Table. Underlying data for this figure can be found in S3 Data. BMAL1, brain and muscle Arnt-like protein-1; BrdU, bromodeoxyuridine; CCD, chronic circadian desynchrony; Cdt1-RFP, chromatin licensing and DNA replication factor 1 tagged with red fluorescent protein; CRY, Cryptochrome; CTL, control; dex, dexamethasone; FITC, fluorescein isothiocyanate; FUCCI, fluorescence ubiquitination-based cell-cycle indicator; GAPDH, glyceraldehyde 3-phosphate dehydrogenase; GFP, green fluorescent protein; *GMNN*, *Geminin*; JL, jet lag; MTT, thiazolyl blue tetrazolium bromide; n.s., not significant; RNA-Seq, RNA sequencing; si-BMAL1, siRNA targeting BMAL1; si-CRY1/2, siRNA targeting both CRY1 and CRY2; si-CTL, control siRNA; siRNA, small interfering RNA; TRITC, tetramethylrhodamine; U2OS, human U2 osteosarcoma.

Consistent with the transcriptome analysis, a trypan blue exclusion assay after CCD revealed a significant increase in cell number in JL samples compared with CTL (Fig 3A).

Importantly, the effect of CCD on molecular cycles and cell number did not depend on the use of dex, as similar data were obtained when forskolin was used as a synchronizing agent (S3 Fig). To determine whether CCD-induced increased cell number was mediated by the molecular clock, we depleted the core clock activator (BMAL1) or repressors (CRY1/CRY2) using transient RNA interference (RNAi) in CTL and JL cells 48 hours before CCD (Fig 3B–3G). Notably, the knockdown of either set of core clock genes significantly compromised the cellular response to CCD (Fig 3B and 3C). Similarly, cell viability, as measured with a thiazolyl blue tetrazolium bromide (MTT)-based cell viability assay, was increased following CCD in control cells stably expressing siRNA targetting green fluoresecent protein (si-GFP) cells but not in U2OS cells stably expressing small interfering RNA (siRNA) targeting BMAL1 (si-BMAL1) (Fig 3D and 3E). Consistent with these results, a bromodeoxyuridine (BrdU) incorporation assay during the first week following CCD revealed a significantly higher number of proliferating cells in JL samples relative to CTL samples in the presence of control siRNA (si-CTL), but the relative effect was reduced by knockdown of a core clock regulator (si-BMAL1, si-CRY1/2) (Fig 3F and 3G). Although BMAL1 can have non-circadian effects, the reduced effect of CCD on cell number with two different clock knockdowns that have opposing roles within the clock (BMAL1 and CRY) supports the idea that the core clockwork contributes to the effects of CCD on cell proliferation.

### Circadian desynchrony targets RB phosphorylation and the G1/S transition to promote proliferation

To explore the distribution of the cell cycle following CCD, we utilized fluorescence ubiquitination-based cell-cycle indicator (FUCCI) imaging, which uses fluorescent probes to demarcate stages of the cell cycle (Cdt1-RFP for G1/S and Geminin-GFP for G2/M). We transduced these probes into the cells during the last day of the CCD schedule and found that both FUCCI sensor–positive cells were significantly increased by the CCD/JL protocol (Fig 3H). However, the number of Cdt1-RFP–expressing cells was significantly higher than that of Geminin-GFP–expressing cells (Fig 3I). Besides the CCD-induced changes in protein levels of the FUCCI probes, mRNA levels of *CDT1* were also higher in the JL cells, relative to CTL, across multiple circadian times, whereas mRNA levels of Geminin (*GMMN*), a posttranslational inhibitor of CDT1, showed little change (Fig 3J). The proto-oncogenic effect of CDT1 involves its up-regulation and hyperactivity in DNA replication in malignant tumor cells or tissues [32,33]. Thus, we speculated that activation of specific cell cycle genes contributed to the enhanced proliferation in response to CCD. Indeed, our transcriptomic analysis showed that genes involved in the G1/S phase transition (*CDK3*, *MYC*, *CCND3*, *CCND1*, and *CDT1*) were highly induced upon CCD, more so than genes that function in other parts of the cell cycle (S, G2, M) (Fig 3K, S4 Fig and S9 Table). These results together suggest that chronic disturbance of cellular rhythms influences the transcriptional network to preferentially activate G1/S phase progression and thereby enhance cell proliferation.

In addition to transcript levels, the G1 to S phase transition is tightly regulated by phosphorylation of a number of cell cycle regulatory proteins [11]. In fact, a key event for G1/S cell cycle progression is phosphorylation of RB by cyclin D-CDK4/6, which leads to activation of the G1/S transition-driving E2 transcription factor (E2F) (S5A and S5B Fig). Several phosphorylation site mapping studies have reported that RB undergoes extensive phosphorylation during the G1/S cell cycle progression [34] (S5A Fig). We sought to examine how CCD affects the phosphorylation status of RB using available phosphorylation site-specific antibodies (pRB-S807/S811, pRB-S795, pRB-S780, pRB-S612; S5A Fig). Western blot analysis using phospho-RB antibodies revealed that CCD induced significantly higher phosphorylation levels at many sites (S807/S811, S795, S780) in RB (S5C and S5D Fig). However, phosphorylation at S612 was not significantly increased. Interestingly, total RB protein levels were also significantly increased in JL cells compared with CTL cells despite similar levels of RB mRNA (*RB1*) in both cell samples (S5E Fig). Thus, the elevated RB protein level may result from increased protein stability mediated by CCD-induced phosphorylation.

Phosphorylation of specific residues of RB at the carboxy terminus by different G1 Cyclin-CDK complexes contributes to its differential function in cell cycle progression and proliferation [34–36]. In this regard, we observed relatively higher phosphorylation at positions S807/S811, compared with the other phosphosites, in response to CCD (S5C Fig). To determine if the regulation of RB phosphosites in U2OS cells is consistent with previous reports in other systems, we used expression constructs encoding wild-type RB, non-phosphorylatable RB with 15 putative CDK sites converted to Ala residues (RB-ΔCDK), and single CDK site monophosphorylatable RB proteins (RB-ΔCDK+S612, RB-ΔCDK+S780, RB-ΔCDK+S807, RB-ΔCDK+S811) (S6 Fig). The expression of the wild-type and phosphosite–modified RB proteins was confirmed by western blot (WB) using phospho-specific antibodies (S6B Fig). FUCCI cell cycle analysis of cells expressing each of these RB constructs revealed that either or both of the S807 and S811-monophosphorylated RB proteins prominently increased G1/S phase cells relative to wild-type, non-phosphorylated, and other single CDK site RB proteins (S6A and S6C Fig). Interestingly, RB lacking all phosphorylation sites (RB-ΔCDK) resulted in more G1/S phase cells than wild-type RB, suggesting that some phosphorylation sites inhibit this transition. In parallel, MTT cell viability analysis also showed significantly enhanced cell proliferation in cells expressing the S807 and S811 monophosphorylated RB proteins alone or in combination (S6D Fig). Indeed, phosphorylation of S807/S811, which alleviates the suppressor function of RB, has been proposed as a representative marker for G1 progression through S phase [37–39]. Moreover, phosphorylation of RB at S807 protects against apoptotic cell death by regulating B-cell lymphoma 2 (Bcl2)-associated X (Bax) activity [40].

To directly assess whether phosphorylation of RB of S807/S811 contributes to cell proliferation upon circadian disturbance, we used C33A cervix carcinoma cells that express a truncated unstable RB protein and so are considered null for RB [41]. We expressed wild-type RB or alanine mutants S807A and S811A of RB (RB-S807A, RB-S811A) in C33A cells and measured cell density with and without CCD (S7A and S7B Fig). As expected, when a tumor suppressor is removed, RB-null cells showed high cell numbers and a very robust response to CCD. C33A cells rescued with wild-type RB showed a smaller but still significant response, but effects of CCD were blunted in C33A cells expressing RB-S807A or RB-S811A (S7C Fig). Together, these data suggest that phosphorylation at S807/S811 plays a unique role in enhancing cell proliferation upon CCD and so likely contributes to the cell proliferation phenotype caused by chronic circadian disturbance.

**Fig 4:**
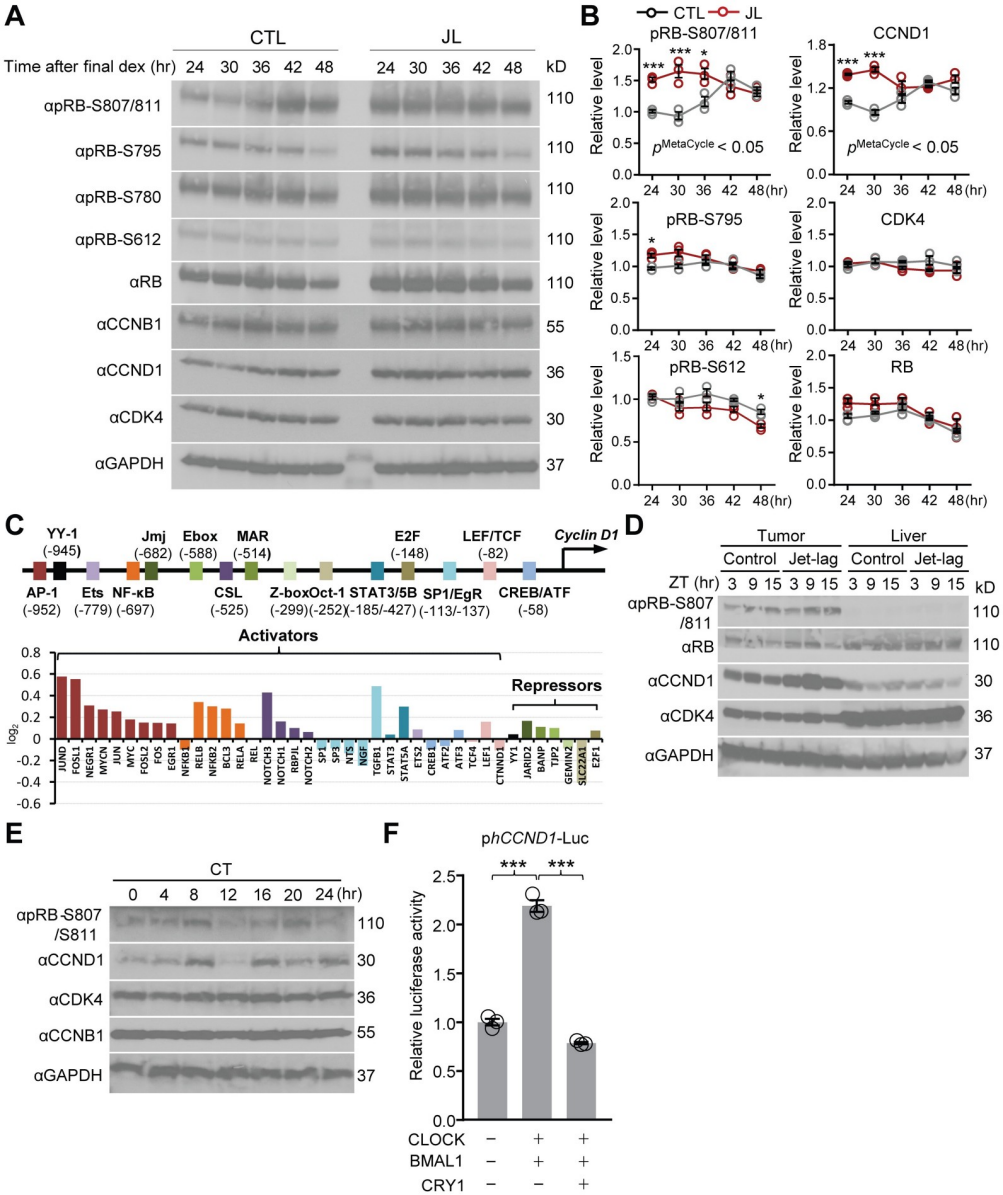
CCD alters rhythmic RB phosphorylation in a cyclin D1–dependent manner. (A) Western blot analysis of phosphorylated RB at multiple sites (pRB-S807/811, pRB-S795, pRB-S780, pRB-S612), total RB, CCNB1, cyclin D1, and CDK4 with the specific antibodies as indicated in the control (CTL) and jet lag (JL) cells collected every 6 hours (24 hours, 30 hours, 36 hours, 42 hours, 48 hours) for 24 hours after the final dex stimulation, as depicted with black arrowheads in Fig 1A. GAPDH (αGAPDH) is loading control. Representative images were taken from *n* = 3 independent experiments. (B) Statistical analysis of the WB data in (A) showing time-dependent variation of protein abundance as indicated in CTL (grey circle) and JL (brown circle) cells. GAPDH was used to normalize protein levels. *p < 0.05, ***p < 0.001; two-way ANOVA and Sidak multiple comparisons test. *p*^MetaCycle^ < 0.05 denotes results of MetaCycle analysis, identifying a 24-hour rhythm in the expression of pRB (S807/S811) (*p* = 0.0481) and CCND1 protein (*p* = 0.006) in CTL cells (grey circle). Data normalized are represented as mean ± SEM from *n* = 3 independent experiments. (C) Schematic representation of the elements of the human *cyclin D1* promoter (upper). Elements of human *cyclin D1* promoter are represented by different colors. The transcriptional start site is identified by a black line and arrow. The data show log_2_ fold-change values for gene expression of signaling-dependent transcriptional activators or repressors that directly target enhancer elements in the *cyclin D1* promoter, as indicated by the corresponding color codes (See S15 Table). (D) Carcinogen (MCA)-induced tumor bearing mice were exposed to a chronic jet-lag schedule (see Materials and methods), following which tumor and liver tissues were harvested from Jet-lag or Control mice at the indicated time points (ZT3, ZT9, ZT15) and subjected to western blot analysis using the indicated antibodies. Representative images from *n* = 3 independent experiments are shown. (E) Immunoblot analysis using liver extracts prepared from mouse liver tissues collected at 4-hour intervals as indicated for 24 hours in constant darkness. Specific antibodies were used for detecting endogenous pRB-S807/811, cyclin D1, CDK4, and CCNB1 proteins, as indicated. Anti-GAPDH (αGAPDH) was used for loading control. Similar results were obtained in two independent experiments. (F) HEK293T cells were transiently transfected with a cyclin D1-Luc reporter construct (p*cyclin D1*(−1748)-Luc) alone or co-transfected with plasmids expressing CLOCK, BMAL1, and CRY1, as indicated. After 24 hours, the cells were lysed and cyclin D1 promoter-driven luciferase activity was measured and normalized with pRL-TK activity. Representative results from three independent experiments performed are shown with the means ± SEM; *n* = 3. ***p < 0.0001, one-way ANOVA and Tukey multiple comparisons test. Underlying data for this figure can be found in S3 Data. AP-1, activator protein 1; BMAL1, brain and muscle Arnt-like protein-1; CCD, chronic circadian desynchrony; CCNB1, cyclin B1; CCND1, cyclin D1; CDK, cyclin dependent kinase; CLOCK, circadian locomotor output cycles protein kaput; CREB/ATF, cAMP response element-binding protein/activating transcription factor; CRY1, Cryptochrome1; CSL, CBF1, Suppressor of Hairless, Lag-1; CT, circadian time; dex, dexamethasone; E-box, enhancer box; Ets, E26 transformation-specific transcription factor; E2F, E2 transcription factor; GAPDH, glyceraldehyde 3-phosphate dehydrogenase; Jmj, jumonji and AT-rich interaction domain containing 2 (Jarid2); LEF/TCF, lymphoid enhancer-binding factor/T-cell factor; Luc, luciferase; MAR, matrix-associated region; MCA, methylcholanthrene; NFKB, nuclear factor kappa-light-chain-enhancer of activated B cells; Oct-1, POU domain, class 2, transcription factor 1; pRB, phosphorylated RB; pRL-TK, renilla luciferase reporter of the HSV-thymidine kinase promoter; RB, retinoblastoma; SP1/EgR, specificity protein 1 (SP1)/early growth response (EgR) transcription factor; STAT3/5B, signal transducer and activator of transcription 3/5B; WB, western blot; YY-1, yin yang 1 transcription factor; Z-box, Z-DNA forming sequence; ZT, zeitgeber time.

Multiple phosphoproteins that function in cell signaling, metabolic, and cell cycle pathways exhibit 24-hour oscillations [42,43]. To determine if RB phosphorylation was affected by CCD in a time-of-day–specific manner, we exposed cells to a CCD schedule and found that phosphorylation at targeted sites of RB, with the possible exception of S612, exhibited a consistent increase across circadian time in JL cells compared with CTL cells (Fig 4A).

Notably, phosphorylation at S807/S811 was found to cycle in CTL cells, but the circadian phase was reversed in JL cells (Fig 4B).

### Cyclin D1 mediates effects of circadian desynchrony on RB phosphorylation

To understand the mechanism underlying the effects of CCD on RB phosphorylation, we examined the expression of cell cycle genes under CCD. Cyclin D1 (CCND1) protein expression displayed increased expression and a reversed cyclic pattern in response to CCD, while other cell cycle molecules (CDK4, cyclin B1 [CCNB1]) were relatively unchanged (Fig 4A and 4B). mRNA expression of cyclin D1 was also persistently induced over most circadian time points in JL cells relative to CTL cells, but this was not seen for other RB pathway components (RB1, CDK4, CDK6) (S8A Fig). These results point to a principal role of cyclin D1 in promoting CDK4/6 kinase activity to drive increased expression and oscillation of RB phosphorylation in response to CCD.

The cyclin D1 gene responds directly to and integrates multiple signaling pathways via its various enhancer elements during the G1/S transition [9]. To address the mechanism responsible for CCD-driven cyclin D1 gene activation, we examined the RNA-Seq data for differences between CTL and JL samples with respect to signaling pathways known to regulate cyclin D1 expression (S8B–S8F Fig, and S10, S11, S12, S13 and S14 Tables). Expression of a large number of key signaling molecules that activate cyclin D1 (wingless/integrated [Wnt], extracellular signal-regulated kinase/mitogen activated protein kinase [ERK/MAPK], phosphatidylinositol 3-kinase/alpha serine/threonine-protein kinase [PI13K/AKT], hippo signaling pathway [HIPPO], G protein-coupled receptor [GPCR]) was up-regulated, whereas most genes that inhibit cyclin D1 were relatively unaffected or down-regulated in response to CCD (S8B–S8F Fig, and S10, S11, S12, S13 and S14 Tables). Correspondingly, several transcriptional activators that respond to such upstream signals and directly target enhancer elements of the cyclin D1 promoter (activator protein 1 [AP-1], nuclear factor kappa B [NFKB], notch signaling pathway [NOTCH]) exhibited up-regulated gene expression relative to repressors, as a result of CCD (Fig 4C, and S15 Table).

To verify CCD-induced activation of cyclin D1 in vivo, we induced a fibrosarcoma in mice using methylcholanthrene (MCA), a potent chemical carcinogen, and exposed the mice to a chronic jet-lag schedule [4]. As described below, tumor growth showed a notably higher rate in jet-lagged mice compared with controls. Western blot analysis revealed that cyclin D1 protein expression as well as RB phosphorylation were markedly increased at multiple time points in tumor tissue, but not in liver tissue, relative to control mice samples assayed in parallel (Fig 4D). This is consistent with previous reports of the cyclin D1-CDK4/6 dependence of RB phosphorylation [39,44]. These data strongly suggest that chronic circadian disturbance promotes an intracellular signaling environment that stimulates cyclin D1 expression in tumors.

Around-the-clock assays of mouse liver extracts at 4-hour intervals showed constitutive expression of CDK4 and CCNB1. Diurnal variations were observed in cyclin D1 expression and RB 807/811 phosphorylation, but were not as robust as seen in cells, perhaps because the adult liver is a non-proliferating tissue (Fig 4E). We also asked if cyclin D1 is a direct target of the clock, and so sought to investigate if a canonical enhancer box (E-box) sequence, located at −588 in the upstream region of the human cyclin D1 gene (Fig 4C), is relevant for clock-mediated expression. To address this, we conducted transcriptional analysis using a luciferase reporter (ph*CCND1*-Luc) in HEK293T cells that are typically used for such assays [22]. We found that cyclin D1 promoter activity was significantly up-regulated by CLOCK/BMAL1 and repressed by CRY1 when co-expressed (Fig 4F). Thus, cyclin D1 is a direct target of the core clock machinery, supported by recent microarray analysis identifying cyclin D1 as a rhythmically expressed gene in most mouse tissues [45]. Taken together, these data suggest that cyclin D1 is a critical component required for rhythmic RB phosphorylation by CDK4/6, mediating circadian coordination or alteration of G1/S phase progression.

### A cyclin D1-CDK4/6 kinase inhibitor has time-of-day–specific effects on tumor growth

**Fig 5:**
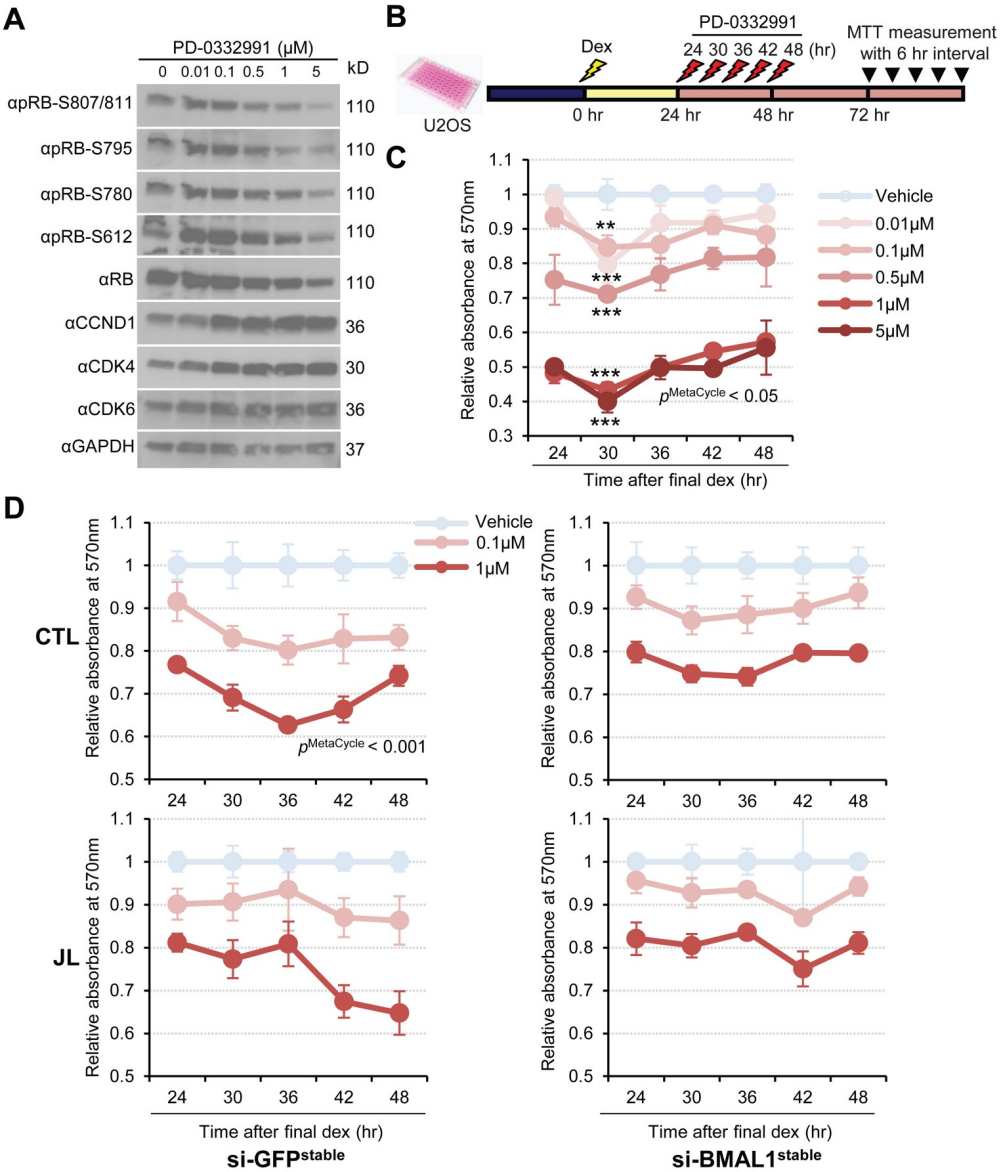
CCD alters time-dependent anticancer activity of PD-0332991, a potent CDK4/6-specific inhibitor, in U2OS cells. (A) Western blot analysis of the dose dependency of PD-0332991 action on RB phosphorylation in U2OS cells. After a 48-hour incubation with increasing doses of PD-0332991, cell extracts were harvested for immunoblot analysis of protein abundances of phosphorylated RBs at multiple sites (pRB-S807/811, pRB-S795, pRB-S780, pRB-S612), total RB, cyclin D1, CDK4, and CDK6 using specific antibodies. Anti-GAPDH (αGAPDH) was used for loading control. (B) Schematic of the experimental schedule to determine time dependence of the antiproliferative effect of PD-0332991 on U2OS cells. After 24 hours of dex (100 nM, yellow bolt) synchronization, the cells were treated with vehicle or PD-033291 (0.01–5 μM, red bolt) at 6-hour intervals over the course of 24 hours and subjected to an MTT cell proliferation assay at the indicated time points 48 hours later. (C) Graph of experimental procedures in (B) to show time-dependent antiproliferative effects of PD-0332991 at various doses. **p < 0.001, ***p < 0.0001; two-way ANOVA and Tukey multiple comparisons test. *p*^MetaCycle^ < 0.05 denotes results of MetaCycle analysis, identifying a 24-hour rhythm in drug sensitivity of cells treated with 0.1 μM PD-0332991 (*p* = 0.0146). Data were normalized to represent the average ± SD; *n* = 3 in all time points. The result is representative of three independent experiments. (D) Twenty-four hours after the final dex stimulation depicted in Fig 1A, Control (CTL) and Jet lag (JL) U2OS cells stably expressing siRNA against GFP (si-GFP^stable^) or BMAL1 (si-BMAL1^stable^) were subject to a time course of vehicle or PD-0332991 (0.1 μM, 1 μM) treatment, and subsequent MTT analysis was performed as described in (B). Data were normalized to represent the average ± SD; *n* = 3 in all time points. *p*^MetaCycle^ < 0.001 denotes results of MetaCycle analysis, identifying a 24-hour rhythm in drug sensitivity of cells treated with 1 μM PD-0332991 (*p* = 0.00019). The result is representative of two independent experiments. Underlying data for this figure can be found in S3 Data. BMAL1, brain and muscle arnt-like protein-1; CCD, chronic circadian desynchrony; CDK, cyclin dependent kinase; dex, dexamethasone; GAPDH, glyceraldehyde 3-phosphate dehydrogenase; GFP, green fluorescent protein; MTT, thiazolyl blue tetrazolium bromide; RB, retinoblastoma; siRNA, small interference RNA U2OS, human U2 osteosarcoma.

The cyclin D1-CDK4/6–RB pathway is a major target for G1/S phase inhibitors in cancer chemotherapy [46]. For instance, palbociclib (PD0332991) is a potent and selective CDK4/6 inhibitor used for the treatment of specific cancers [47]. We found that palbociclib efficiently and dose-dependently blocked RB phosphorylation at several residues, in conjunction with reduced levels of total RB protein, in U2OS cells (Fig 5A).

This supports our prior hypothesis of phosphorylation-dependent stabilization of RB. Conversely, the phospho-RB inhibitor gradually increased protein abundance of cyclin D1 or CDK4/6 dose dependently (Fig 5A). This is consistent with previous reports showing that palbociclib can stabilize cyclin D-CDK4/6 complexes by inducing adaptive responses in mammalian target of rapamycin (mTOR) or phosphatidylinositol 3-kinase/alpha serine/threonine-protein kinase PI3K/AKT pathways [48,49]. Considering the cyclic RB phosphorylation in our data above, we sought to examine circadian kinetics of the antiproliferative effect of palbociclib on dex-synchronized U2OS osteosarcoma cells (Fig 5B). The time course of an MTT cell viability assay revealed that palbociclib exhibited significantly higher antiproliferative activity at 30 hours, relative to other time points, following dex synchronization in both 48-hour and 72-hour drug incubation protocols (Fig 5C, and S9A and S9B Fig). Palbociclib did not show a substantial effect on circadian rhythmicity, as assayed by determining its effect on the *Per2* reporter in U2OS cells, although slightly prolonged rhythms were observed at higher doses, probably due to severe cell growth arrest by the drug (S7C, S7D, S9E and S9F Figs).

To determine whether rhythmic variations in drug efficacy were circadian clock dependent, we subjected U2OS cells stably expressing BMAL1 siRNA (si-BMAL1^stable^), or GFP siRNA as a control (si-GFP^stable^), to CCD and then followed up with the timed pharmacological experiments as above. MTT analysis showed a circadian pattern of drug sensitivity (*p*^MetaCycle^ < 0.05) in control si-GFP^stable^ cells, but the rhythm of drug response was phase reversed in JL GFP^stable^ cells, reflecting altered cycling in these cells (Fig 5D, left panels). Furthermore, rhythmic effects of the inhibitor were abrogated in CTL and JL si-BMAL1^stable^ cells (Fig 5D, right panels), although they were not totally eliminated—likely because siRNA does not produce complete knockdown. These results indicate that the antiproliferative effect of palbociclib, which targets CDK4/6 activity, is regulated by the circadian clock and can be severely altered by rhythm desynchrony.

**Fig 6:**
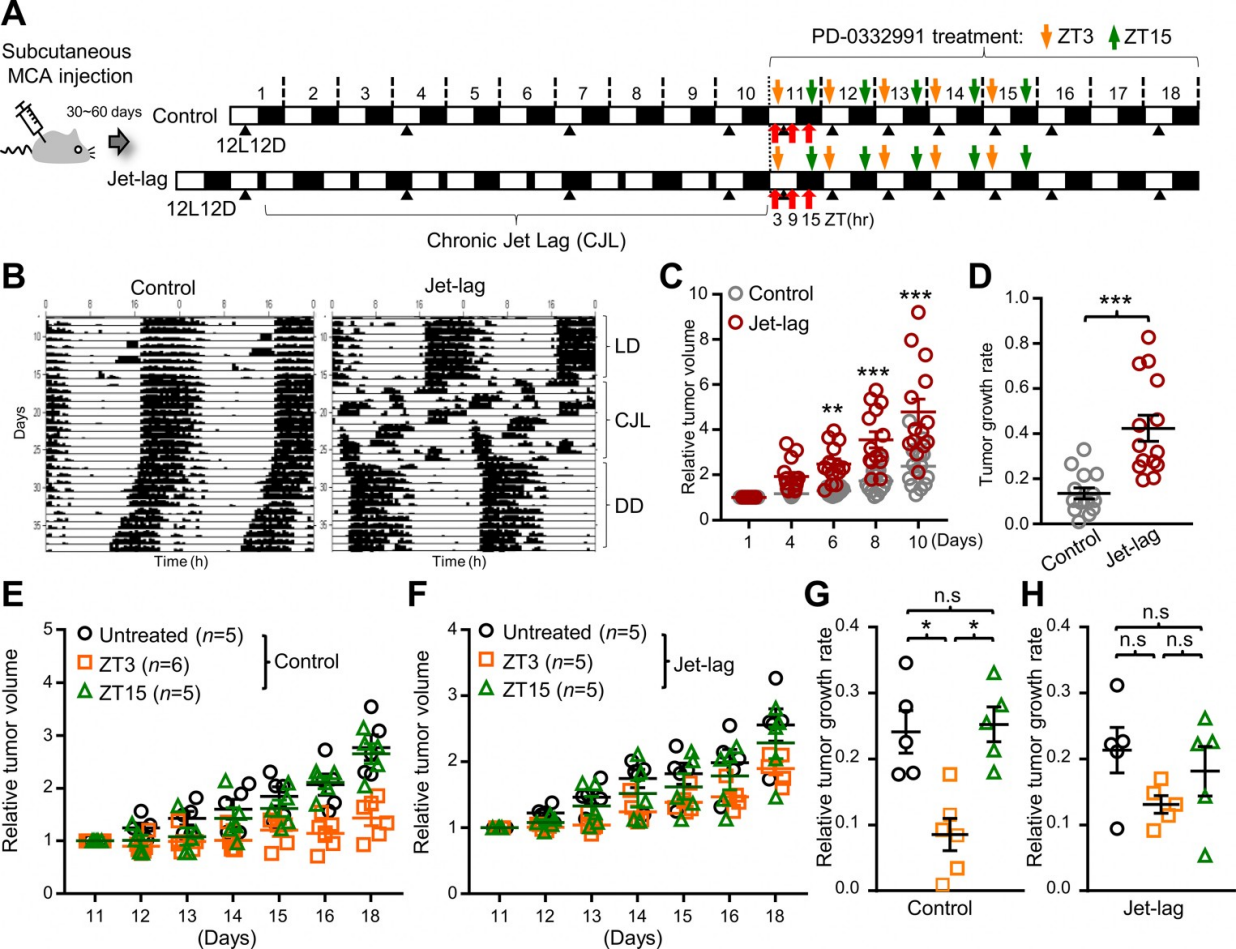
Chronic jet lag alters growth rate of carcinogen (MCA)-induced tumors and time-dependent anticancer activity of PD-0332991 in mice. (A) The experimental schedule for chronic jet lag and palbociclib (PD-0332991) drug treatment. Thirty to sixty days after subcutaneous injection of MCA in mice, the mice were separated into Control and Jet-lag groups for a chronic jet-lag schedule, as depicted. Red arrows indicate sampling of the tumor and liver tissues from both groups of mice killed at the time points (ZT3, ZT9, ZT15) as indicated for further western blot analysis (See Fig 4D). Black arrowheads denote times of tumor measurement. Orange and green arrows indicate oral drug administration of mice at ZT3 and ZT15. Treatment started on day 11 after chronic jet lag (CJL). (B) Representative activity records of running wheel activity in Control and Jet-lag mice. (C) Plots depicting tumor growth in Control (grey circle, *n* = 14) and Jet-lag (brown circle, *n* = 14) mice during CJL. **p < 0.01, ***p < 0.001, two-way ANOVA and Bonferroni multiple comparisons test. Data normalized were derived from *n* = 3 independent experiments. Error bar shown with mean ± SEM. (D) Quantification of relative tumor growth rate calculated from linear regression by fitting a linear equation to observed data in the Control (*n* = 14; grey circle) and Jet lag (*n* = 14; brown circle) mice of (C). ***p < 0.0001, two-tailed Student *t* test. Data normalized were shown with mean ± SEM; *n* = 14 in both groups. (E and F) Time-dependent effects of palbociclib on MCA-induced tumor growth in Control (E) or Jet-lag (F) mice. Tumor growth changed as a function of palbociclib administration time: untreated (black circle), treated at ZT3 (orange square), and treated at ZT15 (green triangle). *n* indicates the number of mice analyzed. Data normalized were shown with mean ± SEM; *n* = 5–6 per group. The result is representative of two independent experiments. (G and F) Quantification of MCA-induced tumor growth rate calculated from linear regression by fitting a linear equation to observed data in Control (G) or Jet-lag mice (F) under the different drug treatment conditions: untreated (black circle), treated at ZT3 (orange squares), and treated at ZT15 (green triangles). *n* = 5–6 mice were analyzed in all groups. *p < 0.05, one-way ANOVA and Tukey multiple comparison test. Data were shown with mean ± SEM. Underlying data for this figure can be found in S3 Data. CJL, chronic jet lag; DD, constant darkness; LD, light-dark; MCA, methylcholanthrene; n.s., not significant; ZT, zeitgeber time.

Based on these findings, we sought to determine if a G1/S cell cycle–specific inhibitor shows time-of-day–specific effects in an in vivo mouse model. To this end, 30–60 days after subcutaneous injection of the MCA carcinogen, mice were subjected to a chronic jet-lag (CJL) protocol, the effects of which were verified by testing the mice for locomotor rhythms (Fig 6A and 6B).

Consistent with a previous study [4], jet-lagged mice (Jet-lag) formed tumors faster than controls (Control) (Fig 6C and 6D). To investigate the time-dependent efficacy of palbociclib, we gave daily oral treatment to Control and Jet lag mice for 5 days at three hours after lights on (zeitgeber time [ZT3]) (morning) or three hours after lights off (ZT15) (night) following the chronic jet-lag schedule (Fig 6A). A significant reduction of tumor growth rate was observed in Control mice receiving the drug at ZT3, but not those treated at ZT15 (Fig 6E and 6G). Moreover, the time-dependent antitumor activity of palbociclib was compromised in Jet lag mice (Fig 6F and 6H). To explore time dependence of this drug with respect to other types of tumors, we induced tumors in mice through subcutaneous injection of B16 mouse melanoma cells, which possess intrinsic circadian function [15]. Interestingly, similar to the results observed in MCA-induced tumors, the inoculated melanoma grew faster in Jet-lag mice than Control during the chronic jet-lag regime (S10A, S10B, S10C and S10D Fig). Furthermore, the melanoma tumors exhibited significantly higher drug sensitivity at ZT3 than ZT15 in Control mice when subjected to daily treatment with palbociclib at these respective time points (S10E and S10G Fig). As with the MCA-induced tumors, time-of-day effects of the drug were severely compromised in Jet-lag mice (S10F and S10H Fig). In conjunction with the cell-based data, our in vivo animal data indicate that circadian regulation of G1/S progression confers time-of-day sensitivity to antitumor agents that act at this step, but the rhythm of sensitivity is lost under conditions of circadian desynchrony.

## Discussion

In this study, we demonstrate the feasibility of a cell-based assay to investigate the impact of circadian desynchrony on a cellular/molecular level. The use of this model allowed us to comprehensively assess impairments in cell physiology induced by a jet-lag protocol, and it revealed a molecular mechanism that is not only relevant for the role of circadian dysregulation in cancer but also provides a molecular target for chronotherapy. Importantly, we show that the mechanism as well as time-of-day effects of a drug targeting the mechanism are manifest in mouse tumor models.

The dex synchronization model is used largely to induce free-running circadian oscillations in mammalian cell lines. However, GCs such as dex are also relevant for circadian synchrony in the organism. Besides photic entrainment driven by the SCN, mammalian body clocks can be directly reset by multiple stimuli such as stress, exercise, and nutrition [50], of which GC hormones released from the adrenal cortex are among the most potent non-photic synchronizers [51]. GC levels exhibit robust daily oscillations, with peak expression in the morning and a trough at night, driven by circadian regulation of GC production by a local clock in the adrenal gland as well as by systemic innervation [52]. GCs mediate circadian responses to various psychosocial stresses [53–55], and thus disruption of GC rhythms by shift work or jet lag could be relevant to pathological conditions [56]. Indeed, disruption of daily GC rhythms is even linked to cancer [57], perhaps related to the inhibitory effects of GC on cell proliferation [58,59]. These critical roles of GC in normal circadian physiology provide an important rationale for the use of dex to impose CCD in a cell-based model.

Notably, in our circadian transcriptome data (Fig 1F), a significant number of genes exhibited circadian cycling in the daily dex-synchronized U2OS cells (*n* = 44), compared with cycling genes estimated in the same cells after a single dex treatment in a previous report (*n* = 7) [60]. This could be primarily due to different experimental and rhythm assessment procedures, for instance, with respect to the measured time points and analysis criteria used in each of the studies. Another possibility for the discrepancy is that the regular rhythmic environment, with daily dex treatment, induced more robust cycling of responsive genes. This is reminiscent of previous circadian gene expression profiling data showing that a large number of genes exhibit diurnal cycling, but not all cycle in free run, nor are all directly clock controlled [45,61]. Given the robust diurnal fluctuation of GC, it is tempting to speculate that our daily dex-synchronization regimen closely mimics physiological cycling in the presence of light–dark cycles.

Malignant cell proliferation and tumor growth is a major pathological consequence of chronic jet lag [7]. Underscoring the link between disrupted clocks and cancer, our unbiased approach, which consisted of characterizing many different cellular functions as well as conducting genome-wide RNA-seq to determine how cell physiology is perturbed by circadian disruption, reveals that cell proliferation, along with concomitant changes in gene expression, is the most highly affected function. Our chronic jet-lag protocol results in a dramatic increase in the ratio of oncogenic genes to tumor suppressor genes. Given the tumor suppressive effect of enhanced circadian function in a recent study [15], we suggest that chronic circadian perturbation tips the transcriptional balance of tumor-progressive and -suppressive genes in favor of promoting tumor survival and proliferation. This explains in part why carcinogen-induced or -injected tumors grow faster under chronic jet lag (Fig 6C and 6D, S10C and S10D Fig) [4]. We note that clock proteins may also directly regulate tumor suppressors and oncogenic proteins [62,63]. However, as our chronic jet-lag experiments reported here are performed with cancer cells and induced tumor tissues, we cannot speak to how a jet-lag paradigm would impact nontransformed cells. Also, because not all cancer cells have functional circadian clocks [64], the proposed mechanism would likely only apply to the subsets of cancers that maintain a clock function.

On the other hand, recent studies have shown that circadian rhythms can be reprogrammed or otherwise affected by tumors or multiple tumor components [65]. For example, lung adenocarcinoma and breast cancer reprogram circadian rhythms in liver metabolism and transcription, respectively [66,67]. Furthermore, cell cycle–related oncogenes (MYC, RAS) were found to affect the circadian clock [68,69]. These findings indicate a dynamic bidirectional relationship between circadian disruption and cancer progression.

Several mitogenic and oncogenic pathways signal to the cell cycle machinery through D-type cyclins [9]. Consistent with this, our transcriptomic analysis reveals extensive up-regulation of upstream signals and downstream transcriptional activators of the cyclin D1 gene in U2OS cells exposed to chronic jet lag (Fig 4C and S8 Fig). More interestingly, cyclin D1 expression was markedly elevated in tumors, but not in other tissue, in chronically jet-lagged mice (Fig 4D). These results suggest that the expression of cell cycle proteins is more sensitive to chronic jet lag in cancerous cells. We note that cyclin D3, another D-type G1 cyclin important for cancer cell survival and progression [70,71], was also up-regulated by CCD in our transcriptomic data (Fig 3K and S4B Fig). Our findings regarding cyclin D corroborate the reported oncogenic role of cyclin D in triggering spontaneous tumors or inducing tumors in response to mitogenic and oncogenic signals [72]. Taken together, these data suggest that cyclin D mediates abnormal tumor proliferation in response to chronic jet lag.

Molecular links between the circadian clock and the cell cycle have been suggested in a few previous studies. For instance, in a murine hepatectomy model, circadian regulation of the G2-M transition was proposed to be driven by clock-controlled expression of Wee1 G2 checkpoint kinase (Wee1), a key cell cycle inhibitor that phosphorylates and thereby inactivates the CDK1–cyclinB1 complex [73]. Timely coordination of the G1/S phase is suggested by circadian transcriptional regulation of p21 and p16, which encode inhibitors of cyclin-dependent kinases, including CDK4/6 [74,75]. In addition, the myelocytomatosis oncogene cellular homolog (Myc) proto-oncogene is thought to be regulated by BMAL1/neuronal PAS domain protein 2 (NPAS2) (a CLOCK paralogue) action on its E-boxes, which may contribute to enhanced tumor growth in Per2 mutant mice in response to DNA damage [76]. Myc can act through p27(Kip1), another multifunctional CDK inhibitor, to facilitate the activation of CDK4/6 [77]. Our in vitro and in vivo data demonstrate that CLOCK/BMAL1 directly regulate cyclin D1, which likely contributes to rhythmic CDK4/6 activity (Fig 4), so multiple circadian mechanisms may converge on CDK4/6. We show also that chronic jet lag alters the phosphorylation of RB, which is the rate-limiting substrate of cyclin D1–CDK4/6 in the cell cycle progression [35]. Importantly, RB phosphorylation is predominantly affected at S807/811, which is a known CDK4/6 site [78]. S807/811 on RB serves as a priming site whose induced phosphorylation releases the tumor-suppressive effect of RB and promotes cell cycle entry, as well as cell survival [37–40]. In this regard, rescue of RB-null cells (C33A) with RB phospho mutants (S807/811A) significantly blunted but did not completely abrogate the enhanced cell proliferation upon CCD (S7 Fig). We speculate that RB-like protein 1 (RBL1, p107), which has structural and functional similarities to RB and is regulated by cyclin D–CDK4/6 to de-repress E2F activity during G1/S cell cycle progression [79], could contribute to enhanced cell proliferation upon chronic circadian disturbance.

Emerging cancer therapeutic efforts target G1/S cell cycle progression, with a focus on the development of selective CDK 4/6 inhibitors [46]. For instance, palbociclib (PD0332991, Ibrance; Pfizer) is a potent oral inhibitor of CDK4/6 initially approved by the United States Food and Drug Administration (FDA) for the treatment of metastatic breast cancer in combination with endocrine therapy [80,81]. Numerous preclinical and clinical studies show that palbociclib has antiproliferative activity in several types of RB-positive tumors, and it is becoming more widely used to treat multiple cancers [47,82]. Despite the growing clinical use of palbociclib and other CDK4/6 inhibitors such as abemaciclib (Lilly) and ribociclib (Novartis), these drugs are typically prescribed without any time-of-day indications. Our in vitro and in vivo chronopharmacological studies demonstrate that the cyclin D1-CDK4/6–RB pathway is a target for chronotherapy, likely because of circadian regulation of the G1/S phase. This pathway could even be a circadian marker for cancer therapy targeting the G1/S phase (Figs 5 and 6).

Pharmacodynamic variability among cancer patients remains a daunting challenge in cancer drug therapy [83]. Our findings strongly suggest that environmental or physiological perturbation of circadian rhythms such as shift work, abnormal sleep timing, or irregular psychosociological stresses can underlie interindividual variability in both cancer growth and response to cancer drugs. Circadian disruption may also be relevant for the chronic sleep loss and depression suffered by many cancer patients following diagnosis and treatment [84]. Given this, it is reasonable to expect that resetting of the body clock by scheduled light exposure, mealtimes, or exercise, alongside a carefully timed chemotherapy regimen, would improve antitumor treatment. Taken together, our study provides a mechanism for tumor susceptibility conferred by circadian dysregulation and highlights the importance of judicious application of cancer chronotherapy.

## Materials and methods

### Ethics statement

All live animal experiments were performed according to protocols (806387) approved by Institutional Animal Care and Use Committee of the University of Pennsylvania in accordance with guidelines set by the NIH.

### Cell culture and reagents

U2OS, HEK293T, or C33A cells purchased from ATCC were cultured in Dulbecco's Modified Eagle Medium (DMEM) supplemented with 10% FBS and 1% penicillin-streptomycin (15140122, Thermo Fisher Scientific, Waltham, MA) at 37 °C under 5% CO_2_. The cells were transfected with siRNAs using Lipofectamine RNAi tranfection reagent (13778075, Thermo Fisher Scientific, Waltham, MA) and DNA plasmids using transfection reagents (E2311, Promega, Madison, WI).

### Plasmids

The plasmids encoding wild-type RB, non-phosphorylatable RB with 15 putative CDK sites converted to Ala residues (RB-ΔCDK), and single CDK site monophosphorylatable RB proteins (RB-ΔCDK+S612, RB-ΔCDK+S780, RB-ΔCDK+S807, RB-ΔCDK+S811) were purchased from Addgene (Cambridge, MA), and were designated as follows: pCMV-HA-hRB-WT (58905), pCMV-HA-hRB-ΔCDK (58906), pCMV-HA-hRB-ΔCDK+S811 (58919), pCMV-HA-hRB-ΔCDK+S807 (58918), pCMV-HA-hRB-ΔCDK+S780 (58915), and pCMV-HA-hRB-ΔCDK+S612 (58914). The other set of plasmids encoding wild-type RB and alanine mutants S807A and S811A of RB (RB-S807A and RB-S811A) were kindly provided by Dr. Nancy A. Krucher at the Department of Biology and Health Science, Pace University. For cyclin D1 promoter analysis, a −1748 human cyclin D1 promoter pGL3 basic (32726) and a −962 human cyclin D1 promoter pGL3 basic (32727) were purchased from Addgene. For luciferase assays, pCMV Sport6 plasmids encoding full-length cDNAs of CLOCK, BMAL1, and CRY1 were obtained from the Mammalian Gene Collection (MGC, Thermo Fisher Scientific, Waltham, MA).

### Bioluminescence recording and data analysis

A total of 1.5 × 10^5^ p*Per2* reporter U2OS cells were seeded per 35-mm dish. After the chronic 100 nM dex (D2915, Sigma, St. Louis, MO) or forskolin (S2449, Selleckchem, Houston, TX) synchronization schedule, as depicted in Fig 1A, the cells were treated with dex every seventh morning, delivered in the medium mentioned above. Real-time bioluminescence of the control and jet-lag cells was monitored using a LumiCycle luminometer (Actimetrics, Wilmette, IL), as previously described [21], and period and amplitude of the luminescence data were determined through the LumiCycle software program (Actimetrics, Wilmette, IL).

### Luciferase assay

HEK293T cells transfected with plasmids encoding proteins as indicated in the figures were assayed for luciferase reporter activity using the luciferin reagent (E2920, Promega, Madison, WI) 24 hours post-transfection according to the manufacturer's protocol.

### Immunoblotting

Immunoblot analysis for U2OS cells was performed as described [85] using the following antibodies: anti-BMAL1 (14020, Cell Signaling, Danvers, MA), CRY1 (A302-614A, Bethyl Laboratories, Montgomery, TX), anti-CRY2 (13997-1-AP, Proteintech, Chicago, IL), anti-RB (9309, Cell Signaling, Danvers, MA), anti-pRB-S807/811(8516, Cell Signaling, Danvers, MA), anti-pRB-S795 (9301, Cell Signaling, Danvers, MA), anti-pRB-S780 (8180, Cell Signaling, Danvers, MA), anti-pRB-S612 (AP3236a, Abgent, San Diego, CA), anti-CCNB1 (#4138, Cell Signaling, Danvers, MA), anti-cyclin D1 (ab134175, Abcam, Cambridge, MA), anti-CDK4 (12790, Cell Signaling, Danvers, MA), anti-CDK6 (14052-1-AP, Proteintech, Chicago, IL). For liver tissue extracts, anti-CDK4 (ab137675, Abcam, Cambridge, MA), anti-RB (ab24, Abcam, Cambridge, MA), and anti-pRB-S807/811 (ABC132, MilliporeSigma, Burlington, MA) were used. Anti-GAPDH (sc25778, Santa Cruz Biotech, Dallas, TX) was used as loading control antibody for both U2OS cell and liver tissue extracts.

### Cell-based assays of proliferation, apoptosis, oxidative stress, metabolism, and proteolysis

Twenty-four hours after the final dex stimulation, as per the experimental schedule depicted in Fig 1A, control and jet-lag cells were harvested and subjected to a cell viability assay with 0.4% tryptopan blue (15250061, Thermo Fisher Scientific, Waltham, MA) to determine cell numbers according to the manufacturer’s protocol. Similarly, after 5 × 10^3^ U2OS cells were seeded per well in 96-well plates, cell viability in control or jet-lag cells following the dex (100 nM) or forskolin (100 nM) synchronization schedule was determined colorimetrically by MTT [3-(4,5-dimethylthiazol-2-yl)-2,5-diphenyl-2H-tetrazolium bromide] using the MTT cell Proliferation assay per the kit protocol (V13154, Thermo Fisher Scientific, Waltham, MA) or alamar blue cell viability reagent (DAL1025, Thermo Fisher Scientific, Waltham, MA). Briefly, prior to addition of the MTT reagent, microplates were subject to the removal of media by decantation followed by the addition of 100 μL of fresh media and 10 μL of freshly prepared MTT reagent. The plates were incubated at 37 °C for 4 hours prior to the addition of 100 μL freshly prepared SDS-HCl solution, followed by mixing with a pipettor. The plates were incubated overnight at 37 °C and absorbance read at 570 nm on the Epoch Microplate Spectrophotometer. The same experimental procedures were performed for cell viability analysis of U2OS cells transfected with plasmids encoding RB and various phosphor-RB mutants, as described above.

To determine S-phase distribution, after 2.5 × 10^4^ U2OS cells were seeded per well of 24-well plates, control and jet-lag cells following the dex (100 nM) synchronization schedule were labeled with 10 μM BrdU (ab142567, Abcam, Cambridge, MA) for 6 hours. Cells were fixed in 4% paraformaldehyde (PFA) (in PBS) for 15 minutes at room temperature, washed three times, and incubated with 0.5% Triton X-100 permeabilization buffer for 20 minutes. The fixed cells were incubated with 1N HCl for 10 minutes on ice, 2N HCL for 20 minutes at room temperature to denature, and neutralized with 0.1 M sodium borate (pH 8.5) for 2 minutes. The cells were incubated with anti-BrdU antibody (ab6326, Abcam, Cambridge, MA) overnight and stained by an Alexa Fluor 568–conjugated secondary antibody (A-11077, Thermo Fisher Scientific, Waltham, MA). The cells were imaged with a fluorescence microscope using TRITC (excitation [Ex.] = 579 nm and emission [Em.] = 604 nm) and DAPI (Ex. = 370 nm and Em. = 460 nm) filter sets with the same light exposure time. Proliferating cells represented by Alexa Fluor 568–stained nuclei were counted as a percentage of all nuclei over 10 microscopic fields.

For measurement of cellular death rate, after 1.5 × 10^5^ U2OS cells were plated per 35-mm dish and exposed to the dex (100 nM) synchronization schedule, control and jet-lag cells were harvested for apoptosis analysis performed according to the Annexin V-FITC Early Apoptosis Detection Kit protocol (6592, Cell Signaling, Danvers, MA). Fluorescence was analyzed using a FACS Calibur (BD Biosciences, San Jose, CA) and FlowJo software (FlowJo, LLC, Ashland, OR).

For measurement of H2O2 level, GSH/GSSG ratio, NADP/NADPH ratio, and protease activity in the control and jet-lag cells seeded in a 96-well plate, luminescence-based assays were performed using H2O2 assay kit (G8820, Promega, Madison, WI), NADP/NADPH assay kit (G9081, Promega, Madison, WI), GSH-Glutathione assay kit (V6911, Promega, Madison, WI), and Proteasome-Chymotrypsin-Like Cell-based assay kit (G8660, Promega, Madison, WI) according to manufacturer's protocols.

### FUCCI cell cycle analysis

At the final dex-containing media change during the chronic desynchronization schedule after 2.5 × 10^4^ U2OS cells were seeded per well of 24-well plates, the control and jet-lag cells were transduced with baculovirus-expressing FUCCI cell cycle sensors (Cdt1-RFP, Geminin-GFP) provided by a FUCCI cell cycle sensor kit (P36237, Thermo Fisher Scientific, Waltham, MA) for 48 hours and were fixed in 4% PFA in PBS for microscopic analysis. They were imaged with a fluorescence microscope using GFP (Ex. = 492 nm and Em. = 514 nm), RFP (Ex. = 579 nm and Em. = 604 nm), and DAPI (Ex. = 370 nm and Em. = 460 nm) filter sets with the same light exposure time. For quantification of the fraction of FUCCI cell cycle indicator–positive cells, the proportion of Geminin-GFP–or Cdt1-RFP–positive cells from the total number of Dapi-stained nuclei (>200) in the CTL or JL cells was averaged from four optical fields scanned with a 20× objective. The same experimental procedures were performed for cell cycle analysis of U2OS cells transfected with plasmids encoding RB and various phosphor-RB mutants, as described above.

### Transfection of siRNA

siRNAs targeting human BMAL1 (GS406), CRY1 (SI02757370), CRY2 (GS1408), cyclin D1 (SI02654540), CDK6 (SI00605052), and CDK4 (SI00299789) were purchased from Qiagen. Transfection of 100 pmol siRNAs per well in U2OS cells was conducted with Lipofectamine RNAi Transfection Reagent (13778075, Thermo Fisher Scientific, Waltham, MA) according to the manufacturer's instructions. Immunoblotting was performed 48 hours after transfection.

### Stable siRNA transgenic cell line generation

U2OS cells were stably transfected with lentiviral particles expressing scrambled GFP siRNA as a control (LVP015-G) or pooled human BMAL1 (ARNTL) siRNA (iV001368) purchased from Applied Biological Materials (Richmond, Canada). The cells were grown with the addition of selection marker (G418; Invitrogen) for 4 weeks. After selection, western blot and chronopharmacological experiments were performed as described.

### RNA extraction and library preparation

After the final dex stimulation in the experimental schedule depicted in Fig 1A, control and jet-lag cells were collected at every 6-hour point (24 hours, 30 hours, 36 hours, 42 hours, 48 hours) for 24 hours (black arrows in Fig 1A). Three replicate dishes of cells were collected at each time point and processed as separate samples. Total RNA was extracted from each sample using the RNA extraction kit (74134, Qiagen, Germantown, MD) according to the manufacturer’s protocol. RNA quality was assessed by Bioanalyzer, and samples with a high RNA integrity number (>8) were used for library construction. For each sample, Illumina sequencing libraries were prepared from 100 ng of total RNA using the TruSeq Stranded mRNA Sample Prep Kit (Illumina, San Diego, CA), according to the manufacturer’s protocol (TruSeq RNA Sample Preparation V2 Guide). Each library was prepared using a unique adapter bar code index to allow for multiplexing. All libraries were pooled together and sequenced across three lanes of an Illumina HiSeq 2000 Sequencer running in single-end mode (1 × 100 bp).

### RNA-Seq analysis

Raw RNA-Seq reads were mapped to the GRCh38 build of the human reference genome using STAR v2.5.3a [86], with the following command line arguments:—outSAMtype BAM Unsorted—outSAMunmapped Within KeepPairs—outFilterMismatchNmax 33—seedSearchStartLmax 33—alignSJoverhangMin 8. STAR was provided with gene models from the Ensembl v90 genome annotation [87]. Note that the three fastq files per library (one for each sequencing lane) were aligned separately. Following alignment, the three BAM files for each library were assigned read groups and merged together using the AddOrReplaceReadGroups and MergeSamFiles commands from Picard Tools v2.7.1 (http://broadinstitute.github.io/picard), respectively. For both commands, BAM files were maintained in unsorted order (i.e., sorted by read ID). The Pipeline of RNA-Seq Transformations (PORT) v0.8.4-beta (https://github.com/itmat/Normalization) was used to perform gene-level normalization and quantification of the aligned data. Raw fastq files and quantification tables are available from the Gene Expression Omnibus database (GSE119668).

To identify genes cycling with a 24-hour period, PORT-normalized gene counts from control and jet-lag samples were processed separately using the meta2d function of the *MetaCycle* R package v1.1 [26]. The meta2d function was run with the following arguments: adjustPhase = "predictedPer", combinePvalue = "fisher", weightedPerPha = FALSE, cycMethod = c("JTK", "LS"), minper = 24, maxper = 24. Genes with significant 24-hour rhythms in expression were identified using a meta2d combined false discovery rate (FDR) cutoff of 0.1. To identify genes with broad DE between the control and jet-lag conditions, PORT-normalized gene counts were analyzed using the *limma* R package v3.34 [88]. Differentially expressed genes (DEGs) were identified using a *limma* FDR cutoff of 0.4. This cutoff was selected to yield a sufficient number of DEGs for Ingenuity Pathway Analysis (IPA; Qiagen, https://www.qiagenbioinformatics.com/products/ingenuity-pathway-analysis/). For IPA core analysis, the entire set of input genes served as the background for the enrichment tests.

### Animal studies

C57BL/6J mice (Jackson Laboratory, Bar Harbor, ME) were housed (≤5/cage) under 12-hour light–12-hour dark conditions with food and water available ad libitum. Male mice (2–3 months old) were used in all experiments. After acclimation with standard lighting conditions of LD 12:12, with lights on from 5 AM (Zeitgeber time 0) to 5 PM (Zeitgeber time 12) for 2 weeks, the mice were injected with 1 × 10^6^ B16-10 cells subcutaneously along the right flank and separated into control and chronic jet-lag groups. For MCA-induced tumors, mice were injected subcutaneously along the right flank with 400 μg of 3-MCA in peanut oil (213942, Sigma, Louis, MO). After 30–60 days of the injection, the mice were separated into control and chronic jet-lag groups. The chronic jet-lag mice underwent repeated 8-hour advances of the light–dark cycle every 2 days for 11 days, while the control mice further remained in the LD 12:12 lighting regimen. Their locomotor activity was monitored as described [89]. Tumor growth was measured with a digital caliper three times per week. Tumor weight was computed as follows: (length × width^2^)/2. In all animal experiments, mice were euthanized when the tumor exceeded 20 mm in diameter (approximately a volume of 3,500 mm^3^). Tumor and liver tissues were collected at ZT3, ZT9, ZT15 to measure circadian changes of RB phosphorylation and expression of cell cycle proteins with western blot analysis, as described.

### In vitro and in vivo chronopharmacology

For pharmacological inhibition of CDK4/6 activity in U2OS cells, palbociclib hydrochloride (PD-0332991 HCL) (S1116, Selleckchem, Houston, TX) was used in various experimental settings as described (Fig 5). For in vivo animal studies, palbociclib was dissolved in 50 mmol/L sodium lactate, pH 4, and administered by oral gavage daily (120 mg/kg) at ZT3 and ZT15 after measurable tumors were formed following the chronic jet-lag schedule, as described [90]. For evaluation of antitumor activity of the drug, tumor growth was measured with a digital caliper daily during drug administration. Additional details for each experiment are given in the Fig 6 legend.

### Statistical analysis

All statistical tests used in this study were completed with Prism7 GraphPad Software. For making multiple comparisons, we used one-way or two-way ANOVA followed by Bonferroni, Sidak, and Tukey multiple comparisons tests. For comparing the average of two means, we used the Student *t* test (two-tailed paired or unpaired) to reject the null hypothesis (*p* < 0.05).

## Supporting information

S1 Fig Short-term circadian desynchrony delays and dampens cellular rhythms. (A) Schematic of the experimental schedule. U2OS cells stably expressing *Per2* promoter-driven destabilized luciferase (*pPer2-dLuc*) were treated with media containing 100 nM dex every 24 hours for control (Control; CTL) or repeated 8-hour advances of the daily cycle every 2 days for jet lag (Jet lag; JL) for 6 days, as indicated by yellow arrows. After the CTL and JL schedule, the reporter cells were subjected to real-time recording of bioluminescence activity as indicated by red arrows. (B) Bioluminescence recordings of dex-synchronized cells with CTL or JL schedule described in (A). The data are plotted as results of three cultured dishes for each of the CTL and JL conditions (CTL, black; JL, brown). Red arrow indicates the start of the bioluminescence measurement for CTL and JL cells following the final dex treatment. The yellow arrows indicate the dex stimulations every week (1 week, 2 weeks, 3 weeks, 4 weeks) for a month. (C) The bioluminescence recording data in (B) were detrended by a 24-hour moving average subtraction. (D and E) Period (D) and amplitude (E) analysis of circadian bioluminescence data of CTL (grey circle) and JL (brown circle) cells in (B) and (C) for the first week (1 week). The data presented are the means ± SEM; *n* = 3 (*p < 0.05, by two-tailed Student *t* test). (F) The estimated time lags for the onset of the first peak of rhythms (phase) in CTL (grey circle) and JL (brown circle) samples in the first week after the dex-synchronization schedule. The data presented are the means ± SEM; *n* = 3 (***p < 0.0001, by two-tailed Student *t* test). Representative data from *n* = 3 independent experiments are shown. Underlying data are provided in S3 Data. CTL, control; dex, dexamethasone; JL, jet lag; n.s., not significant; *Per2*, *Period2* U2OS, human U2 osteosarcoma.
(TIF)

S2 Fig Effect of CCD on different physiological parameters in U2OS cells. (A to E) Twenty-four hours after the final dex stimulation, as per the experimental schedule depicted in Fig 1A, control (CTL: grey) and jet lag (JL: brown) cells were harvested and subjected to luminescence-based assays for measurement of H2O2 levels (A), GSH/GSSG ratio (B), NADP/NADPH ratio (C), and proteasome activity (D) or Annexin V-FITC early apoptosis assay, followed by flow cytometric analysis (E) according to the manufacturer's protocols. The data presented (A to D) are the means ± SD; *n* = 3 in all groups. Underlying data are provided in S3 Data. CCD, chronic circadian desynchrony; CTL, control; dex, dexamethasone; FITC, fluorescein isothiocyanate; GSH/GSSG, glutathione/glutathione disulfide; JL, jet lag; U2OS, human U2 osteosarcoma.
(TIF)

S3 Fig Effect of CCD induced by forskolin on cellular rhythms and proliferation. (A) Bioluminescence recordings of 100 nM forskolin (Fsk)-synchronized cells with a control (CTL) or jet lag (JL) schedule as described in Fig 1 A. The data are plotted as results of three cultured dishes for each of the CTL and JL conditions (CTL-Fsk, black; JL-Fsk, brown). (B) The bioluminescence recording data in (A) were detrended by a 24-hour moving average subtraction. Period (C) and amplitude (D) analysis of circadian bioluminescence data of CTL (grey circles) and JL (brown circles) cells in (A) and (B). The data presented are the means ± SEM, *n* = 3 (*p < 0.05, by two-tailed Student *t* test). (E) The estimated time lags for the onset of the first peak of rhythms (phase) in CTL (grey circles) and JL (brown circles) samples following a Fsk-synchronization schedule. The data presented are the means ± SEM; *n* = 3 (**p < 0.01, by two-tailed Student *t* test). (F) Twenty-four hours after the final Fsk stimulation, as per the experimental schedule depicted in Fig 1A, CTL (grey circles) and JL (brown circles) were harvested and subjected to the alamar blue cell viability assay to determine cell proliferation. *p < 0.05, two-tailed Student *t* test. Data are presented as mean ± SD; *n* = 12 samples. Raw data are provided in S3 Data. CCD, chronic circadian desynchrony; Fsk, forskolin; n.s., not significant.
(TIF)

S4 Fig Effect of CCD on the expression of cell cycle genes. (A) Heat map displaying expression patterns of well-characterized cell cycle genes in control and jet lag cells. Genes are grouped by their associated cell cycle phases (G1/S, S, G2, G2/M). Color is scaled by calculating z-scores from normalized RNA-seq read counts within each row. (B, C, D) RNA-seq expression traces from control (CTL; black) and jet lag (JL; brown) samples for representative genes specific to (B) G1/S and (C) G2/M phases of the cell cycle, and (D) cyclin-dependent kinase inhibitor genes (CDKIs). See S9 Table. CCD, chronic circadian desynchrony; CDKI, cyclin-dependent kinase inhibitor gene; CTL, control; JL, jet lag; RNA-Seq, RNA sequencing.
(TIF)

S5 Fig CCD increases RB phosphorylation at sites targeted by cyclin-dependent kinases. (A) Schematic representation of CDK phosphorylation sites in human RB. Position of the consensus Cdk phosphorylation sites in relation to the RB protein is indicated. The A and B domains of the small pocket and large pocket and the carboxyl terminus are indicated. (B) Schematic representation of the cyclin D1-CDK4/6 and/or cyclin E-CDK2 phosphorylation sites in RB required for G0/G1/S phase transition. Complexes involved in this transition are also indicated. Phosphorylation sites (pRB-S807/811, pRB-S795, pRB-S780, and pRB-S612) assayed in subsequent western blot analysis of RB phosphorylation status are highlighted in bold. (C) Western blot (WB) analysis of total RB or phospho-RB proteins (pRB-S807/811, pRB-S795, pRB-S780, pRB-S612), with specific antibodies as indicated in control (CTL) and jet lag (JL) cells 24 hours after the final dex stimulation, as per the experimental schedule depicted in Fig 1A. Anti-GAPDH (αGAPDH) was used for loading control. (D) Statistical analysis of WB data in (C) showing the total or phosphorylated RB proteins at multiple sites as indicated (*p < 0.05, **p < 0.01, ***p < 0.001 by two-way ANOVA and Bonferroni multiple comparisons test). Data normalized are represented as mean ± SD from *n* = 3 independent experiments. CTL (grey bar); JL (brown bar). (E) Comparison of *RB1* expression profiles in CTL (grey bar) and JL (brown bar) cells from RNA sequencing data. n.s., *p* > 0.05. Data normalized are shown with means ± SD; *n* = 3. Underlying data are provided in S3 Data. CCD, chronic circadian desynchrony; CDK, cyclin dependent kinase; CTL, control; dex, dexamethasone; JL, jet lag; n.s., not significant; P, phosphorylation; pRB, phospho-RB protein; RB, retinoblastoma; WB, western blot.
(TIF)

S6 Fig RB phosphorylation at S807/811 promotes G1/S phase progression and cell proliferation in U2OS cells. (A) Forty-eight hours after transfection of the control (Sport6-CTL), the intact RB (RB-WT), ΔCdk RB (RB-ΔCDK), and RB-single-Cdk site constructs in U2OS cells, the cells were transduced with baculovirus-expressing FUCCI cell cycle sensors (Cdt1-RFP for G1/S, Geminin-GFP for G2/M) for 48 hours and were fixed for microscopic analysis. Numbering indicates single Cdk site location on RB. Representative images were captured by fluorescence imaging microscopy using specific filter sets for FITC (grey green; Geminin-GFP), TRITC (red; Cdt-RFP), and DAPI (blue; nuclei). (B) Immunoblot of the intact RB (RB-WT), ΔCdk RB (RB-ΔCDK), and RB-single-Cdk site proteins expressed in U2OS cells using the antibodies indicated. GAPDH (αGAPDH) is loading control. (C) Quantification of the fraction of FUCCI cell cycle indicator–positive cells in the image data shown in (A). Proportion of Geminin-GFP (grey green bar)–or Cdt1-RFP (red bar)–positive cells from the total number of Dapi-stained nuclei (>250) in each of the image panels indicated were averaged from four optical fields scanned with a 20× objective. *p < 0.05, ***p < 0.001 (two-way ANOVA and Tukey multiple comparison test). Data were normalized and represented as mean ± SD; *n* = 4. The result is representative of three independent experiments. (D) Forty-eight hours after transfection of control (Sport6-CTL), intact RB (RB-WT), ΔCdk RB (RB-ΔCDK), and RB-single-Cdk site constructs as indicated in U2OS cells, the cells were incubated in the changed media for 48 hours followed by MTT assay for evaluation of cell proliferation. **p < 0.005, ***p < 0.0001, two-tailed *t* test. Data were normalized and represented as mean ± SD; *n* = 3. The result is representative of three independent experiments. Underlying data are provided in S3 Data. Cdk, cyclin-dependent kinase; Cdt1-RFP, chromatin licensing and DNA replication factor 1 tagged with red fluorescent protein; FITC, fluorescein isothiocyanate; FUCCI, fluorescence ubiquitination-based cell-cycle indicator; GFP, green fluorescent protein; MTT, thiazolyl blue tetrazolium bromide; n.s., not significant; RB, retinoblastoma; RB-ΔCDK, non-phosphorylatable RB with 15 putative CDK sites converted to Ala residues; RB-WT, intact RB; Sport6-CTL, cytomegalovirus promoter driven Sport6 (pCMV-Sport6) control vector; TRITC, tetramethylrhodamine; U2OS, human U2 osteosarcoma.
(TIF)

S7 Fig RB phosphorylation at S807/811 mediates enhanced cell proliferation upon circadian desynchronization in C33A cells. (A) Schematic of the experimental schedule. Forty-eight hours after transfection of plasmids expressing control (CTL), wild-type RB (RB-WT), or alanine mutants S807A and S811A of RB (RB-S807A, RB-S811A) in C33A cells, the cells were treated with media containing 100 nM dex every 24 hours for control (CTL) or repeated 8-hour advances of the daily cycle every 2 days for jet-lag (JL), for 6 days, as indicated by yellow arrows. (B) Immunoblot of RB-WT or alanine mutants S807A and S811A of RB (RB-S807A, RB-S811A) expressed in C33A cells using the antibodies indicated. Actin (αActin) is loading control. Note that the RB-807/811 antibody only recognizes the phosphor-807 site. (C) Alamar blue cell viability measurements of CTL and JL cells after the circadian desynchronization schedule are as indicated with red arrows in (A). *p < 0.05, ***p < 0.001; two-way ANOVA and Tukey multiple comparisons test. Data are shown as means ± SD; *n* = 12 per group. Raw data are provided in S3 Data. CTL, control; JL, jet lag; RB, retinoblastoma; RB-WT, wild-type RB.
(TIF)

S8 Fig Circadian desynchrony activates multiple signaling pathways that drive cyclin D1–mediated cell cycle progression. (A) Comparison of *cyclin D1*, *RB1*, *CDK4*, and *CDK6* mRNA expression profiles from RNA sequencing data in control (CTL; grey circle) and jet lag (JL; brown circle) cells collected every 6 hours, as indicated, for 24 hours following the chronic desynchronization schedule depicted in Fig 1A. *p < 0.05, **p < 0.005; two-way ANOVA with Bonferroni multiple comparisons test. Data are shown with the means ± SEM; *n* = 3 in all time points. (B-F) Log_2_ fold-change values for gene expression of key activators, mediators, or repressors of (B) Wnt, (C) ERK/MAPK, (D) PI3K/AKT, (E) Hippo, (F) GPCR signaling pathways. Fold-change values calculated using RNA sequencing data from CTL and JL cells. Schematics for cellular pathways known to regulate cyclin D1 (CCND1) or pathway target gene (PTG) expression for G0/G1/S phase cell cycle progression are included as panel insets. Colors of bars in fold-change graphs correspond to associated components in the pathway diagrams. See S10, S11, S12, S13 and S14 Tables. CCND1, cyclin D1; CTL, control; dex, dexamethasone; ERK/MAPK, extracellular signal-regulated kinase/mitogen activated protein kinase; GPCR, G protein-coupled receptor; Hippo, hippo signaling pathway; JL, jet lag; PI3K/AKT, phosphatidylinositol 3-kinase/alpha serine/threonine-protein kinase; PTG, pathway target gene; RB, retinoblastoma; RB-WT, wild-type RB; Wnt, wingless/Integrated.
(TIF)

S9 Fig A CDK4/6 inhibitor (PD-0332991) has time-of-day–specific effects on cell proliferation without substantially affecting the circadian clock. (A) Schematic of the experimental schedule to determine time dependence of the antiproliferative effect of PD-0332991 on U2OS cells. (B) After 24 hours of dex (100 nM, yellow bolt) synchronization, the cells were treated with vehicle or PD-033291 (0.01–5 μM, red bolt) at 6-hour intervals over the course of 24 hours and subjected to an MTT cell proliferation assay 72 hours later (see indicated time points). Drug doses used are indicated with graded colors. *p < 0.001, ***p < 0.0001; two-way ANOVA and Tukey multiple comparisons test. The data presented are the means ± SD; *n* = 3 in all groups. (C) Bioluminescence recordings of the *Per2* promoter (pPer2-dsLuc) luciferase rhythm after treatment of U2OS cells with 100 nM dex plus different doses of PD-0332991, as indicated with graded colors (0.01–5 uM). The data were plotted from the results of three cultured dishes for each of the drug doses. (D) Detrended data of (C) with a 24-hour moving average subtraction. (E and F) Period (E) and amplitude (F) analysis of the circadian bioluminescence data. Drug doses used are indicated with graded colors. ***p < 0.0001, one-way ANOVA and Dunnett multiple comparison test. The data presented are the means ± SD; *n* = 3 in all groups. Underlying data are provided in S3 Data. CDK, cyclin dependent kinase; dex, dexamethasone; MTT, thiazolyl blue tetrazolium bromide; n.s., not significant; *Per2*, *Period2*; U2OS, human U2 osteosarcoma.
(TIF)

S10 Fig Chronic jet lag alters growth rate of a melanoma tumor and time-dependent anticancer activity of PD-0332991 in mice. (A) The experimental schedule for chronic jet lag and palbociclib (PD-0332991) drug treatment. The red arrow indicates subcutaneous injection of B16 mouse melanoma cells (1 × 10^6^). Black arrowheads denote times of tumor measurement. Orange and green arrows indicate oral drug administration of mice at ZT3 and ZT15. Treatment started on day 11 after tumor inoculation. (B) Representative activity records of running wheel activity in Control and Jet-lag mice. The red arrow indicates B16 melanoma injection. (C) Plots depicting tumor growth in Control (grey square, *n* = 15) and Jet-lag (brown square, *n* = 14) mice during chronic jet lag. Red arrow indicates B16 melanoma injection. (D) Quantification of melanoma tumor growth rate calculated from linear regression by fitting a linear equation to observed data in Control (*n* = 3; grey square) and Jet-lag (*n* = 4; brown square) mice of (C). *p < 0.05, two-tailed and paired Student *t* test. Data normalized were shown with mean ± SEM. (E and F) Time-dependent effects of palbociclib on melanoma tumor growth in Control (E) or Jet-lag (F) mice. Tumor growth changed as a function of palbociclib administration time; untreated (black circle), treated at ZT3 (orange square), treated at ZT15 (green triangle). *n* indicates number of mice analyzed. Data normalized were shown with mean ± SEM; *n* = 3–4 per group. The result is representative of two independent experiments. (G and H) Quantification of melanoma tumor growth rate calculated from linear regression by fitting a linear equation to observed data in Control (G) or Jet-lag mice (H) under the different drug treatment conditions; untreated (black circle), treated at ZT3 (orange square), or treated at ZT15 (green triangle). *n* = 3–4 mice were analyzed per group. **p < 0.001, one-way ANOVA and Tukey multiple comparison test. Data were shown with mean ± SEM. Underlying data are provided in S3 Data. n.s., not significant; ZT, zeitgeber time.
(TIF)

S1 Table Top cyclers from meta2d analysis of control samples. (XLSX)

S2 Table Meta2d results from jet-lag samples for genes in S1 Table. (XLSX)

S3 Table Top eight genes from limma-voom DE analysis of all control samples versus jet-lag samples. DE, differential expression.
(XLSX)

S4 Table Fold-change analysis of gene expression of oncogenes and tumor suppressor genes based on time course RNA-Seq data of control and jet lag cells. RNA-Seq, RNA sequencing.
(XLSX)

S5 Table Fold-change analysis of gene expression of RAS genes based on time course RNA-Seq data of control and jet lag cells. RAS, retinoic acid syndrome; RNA-Seq, RNA sequencing.
(XLSX)

S6 Table Fold-change analysis of gene expression of EGF-EGFR genes based on time course RNA-Seq data of control and jet lag cells. EGF-EGFR, epidermal growth factor-epidermal growth factor receptor; RNA-Seq, RNA sequencing.
(XLSX)

S7 Table Fold-change analysis of gene expression FGF-FGFR genes based on time course RNA-Seq data of control and jet lag cells. FGF-FGFR, fibroblast growth factor-fibroblast growth factor receptor; RNA-Seq, RNA sequencing.
(XLSX)

S8 Table Fold-change analysis of gene expression of VEGF-VEGFR genes based on time course RNA-Seq data of control and jet lag cells. RNA-Seq, RNA sequencing; VEGF-VEGFR, vascular endothelial growth factor-vascular endothelial growth factor receptor.
(XLSX)

S9 Table Fold-change analysis of gene expression of cell cycle genes based on time course RNA-Seq data of control and jet lag cells. RNA-Seq, RNA sequencing.
(XLSX)

S10 Table Fold-change analysis of gene expression of Wnt pathway genes based on time-course RNA-Seq data of control and jet lag cells. RNA-Seq, RNA sequencing; Wnt, wingless/integrated.
(XLSX)

S11 Table Fold-change analysis of gene expression of ERK/MAP kinase pathway genes based on time course RNA-Seq data of control and jet lag cells. ERK/MAP, extracellular signal-regulated kinase-mitogen activated protein kinase; RNA-Seq, RNA sequencing.
(XLSX)

S12 Table Fold-change analysis of gene expression of PI3K-AKT pathway genes based on time course RNA-Seq data of control and jet lag cells. PI3K-AKT, phosphatidylinositol 3-kinase/alpha serine/threonine-protein kinase; RNA-Seq, RNA sequencing.
(XLSX)

S13 Table Fold-change analysis of gene expression of HIPPO pathway genes based on time course RNA-Seq data of control and jet lag cells. HIPPO, hippo signaling pathway; RNA-Seq, RNA sequencing.
(XLSX)

S14 Table Fold-change analysis of gene expression of GPCR genes based on time course RNA-Seq data of control and jet lag cells. GPCR, G protein-coupled receptor; RNA-Seq, RNA sequencing.
(XLSX)

S15 Table Fold-change analysis of gene expression of transcriptional regulators of cyclin D1 promoter elements based on time course RNA-Seq data of control and jet lag cells. RNA-Seq, RNA sequencing.
(XLSX)

S1 Data Time course RNA-Seq data of control and jet lag cells. RNA-Seq, RNA sequencing.
(XLSX)

S2 Data Results from limma-voom DE analysis of all control versus jet lag samples. DE, differential expression.
(XLSX)

S3 Data Excel spreadsheet containing, in separate sheets, the underlying numerical data and data analysis for figure panels. (XLSX)

## Abbreviations

AKT: alpha serine/threonine-protein kinase serine/threonine kinase
ANXA1: annexin A1
AP-1: activator protein 1
BAX, B: cell lymphoma 2-associated X
BMAL1: brain and muscle arnt-like protein-1
BRCA1: breast cancer type 1 susceptibility protein
CCD: chronic circadian desynchrony
CCNB1: cyclin B1
CCND1: cyclin D1
CCND3: cyclin D3
CD36: cluster of differentiation 36
CDK: cyclin dependent kinase
Cdt1: chromatin licensing and DNA replication factor 1
CLOCK: circadian locomoter output cycles protein kaput
Cry: Cryptochrome
CTL: control
DE: differential expression
DEG: differentially expressed gene
dex: dexamethasone
DHX58: DEXH (Asp-Glu-X-His) box polypeptide 58
DMEM: Dulbecco's Modified Eagle Medium
E2F: E2 transcription factor
EGF: epidermal growth factor
EGFR: epidermal growth factor receptor
Em.: emission
ERBB2: erbb2 receptor tyrosine kinase 2
ERK: extracellular signal-regulated kinase
Ex.: excitation
EXOC3L2: exocyst complex component 3-like protein 2
FDA: United States Food and Drug Administration
FDR: false discovery rate
FGF: fibroblast growth factor
FGFR: fibroblast growth factor receptor
FITC: fluorescein isothiocyanate
FUCCI: fluorescence ubiquitination-based cell-cycle indicator
GAPDH: glyceraldehyde 3-phosphate dehydrogenase
GC: glucocorticoid
GPCR: G protein-coupled receptor
GSH: glutathione
GSSG: glutathione disulfide
HIPPO: hippo signaling pathway
HMGA1: high mobility group AT-hook 1
IPA: Ingenuity Pathway Analysis
JAG2: jagged 2
JL: jet lag
JUN: jun oncogene
JUND: Jun proto-oncogene related gene d
MAFA: musculoaponeurotic fibrosarcoma oncogene A
MAFB: musculoaponeurotic fibrosarcoma oncogene B
MAPK: mitogen activated protein kinase
MCA: methylcholanthrene
mTOR: mammalian target of rapamycin
MTT: thiazolyl blue tetrazolium bromide
Myc: myelocytomatosis oncogene cellular homolog
NF1: neurofibromin 1
NFKB: nuclear factor kappa B
NOTCH: notch signaling pathway
NPAS2: neuronal PAS domain protein 2
PCSK1N: proprotein convertase subtilisin/kexin type 1 inhibitor
*Per*: *Period*
*Per2*: *Period2*
PFA: paraformaldehyde
PI3K: phosphatidylinositol 3-kinase/alpha serine/threonine-protein kinase
PORT: Pipeline of RNA-Seq Transformations
RAS: retinoic acid syndrome
RB: retinoblastoma
RBL1: RB-like protein 1
RNA-Seq: RNA sequencing
SCN: suprachiasmatic nucleus
SH3D21: SRC homology domain-containing protein 21
si-BMAL1: siRNA targeting BMAL1
si-CTL: control siRNA
TRITC: tetramethylrhodamine
U2OS: human U2 osteosarcoma
VEGF: vascular endothelial growth factor
VEGFR: vascular endothelial growth factor
WB: western blot
Wee1: Wee1 G2 checkpoint kinase
Wnt: wingless/integrated
ZT: zeitgeber time
