# Supplementary figures and images for: G1/S cell cycle regulators mediate effects of circadian dysregulation on tumor growth and provide targets for timed anticancer treatment
Source: PLoS Biol. 2019 Apr 30;17(4):e3000228. doi: 10.1371/journal.pbio.3000228 (PMC6490878; doi:10.1371/journal.pbio.3000228)

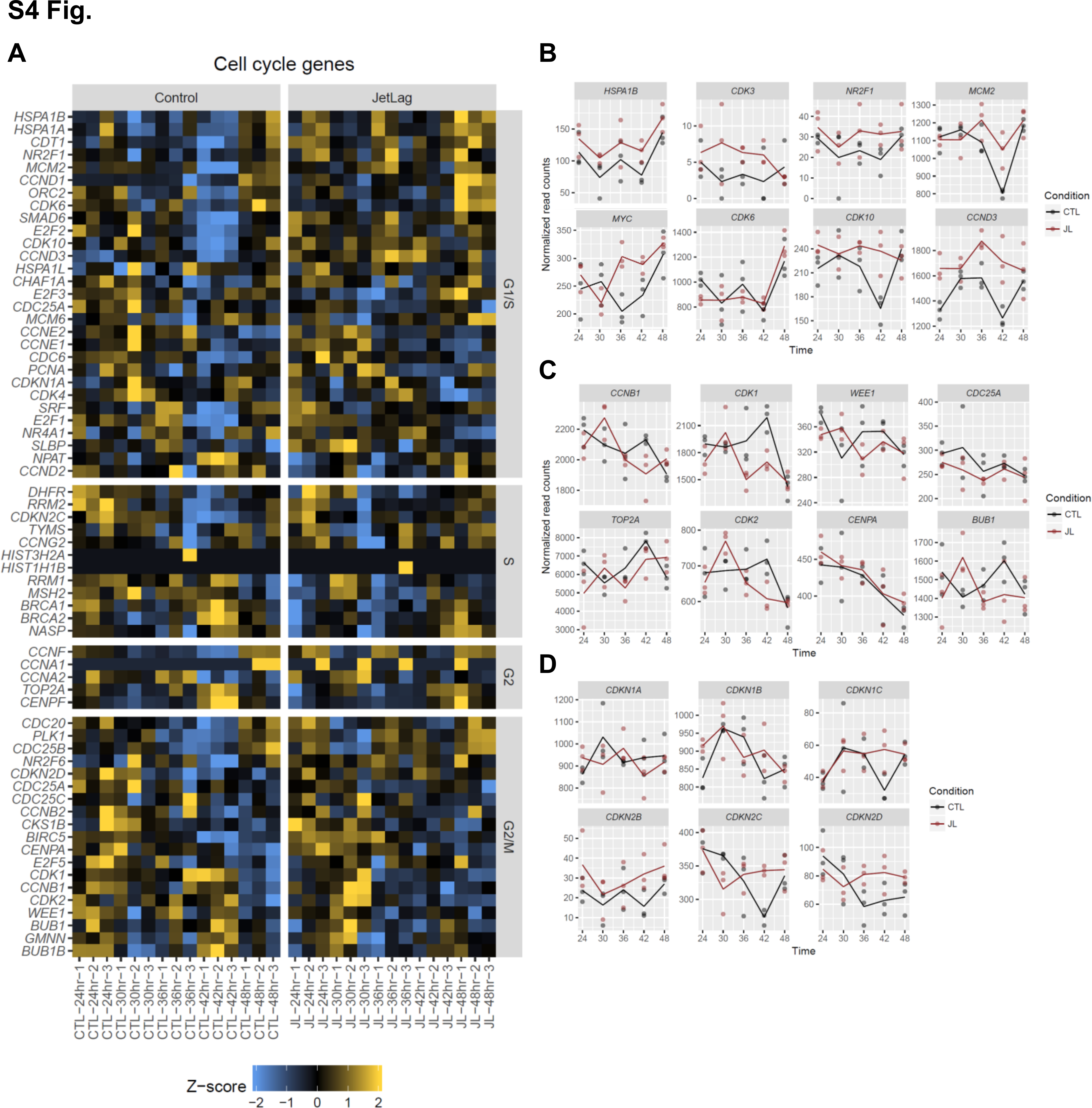

Supplement: S4 Fig — (A) Heat map displaying expression patterns of well-characterized cell cycle genes in control and jet lag cells. Genes are grouped by their associated cell cycle phases (G1/S, S, G2, G2/M). Color is scaled by calculating z-scores from normalized RNA-seq read counts within each row. (B, C, D) RNA-seq expression traces from control (CTL; black) and jet lag (JL; brown) samples for representative genes specific to (B) G1/S and (C) G2/M phases of the cell cycle, and (D) cyclin-dependent kinase inhibitor genes (CDKIs). See S9 Table. CCD, chronic circadian desynchrony; CDKI, cyclin-dependent kinase inhibitor gene; CTL, control; JL, jet lag; RNA-Seq, RNA sequencing. (TIF) [file pbio.3000228.s004.tif]

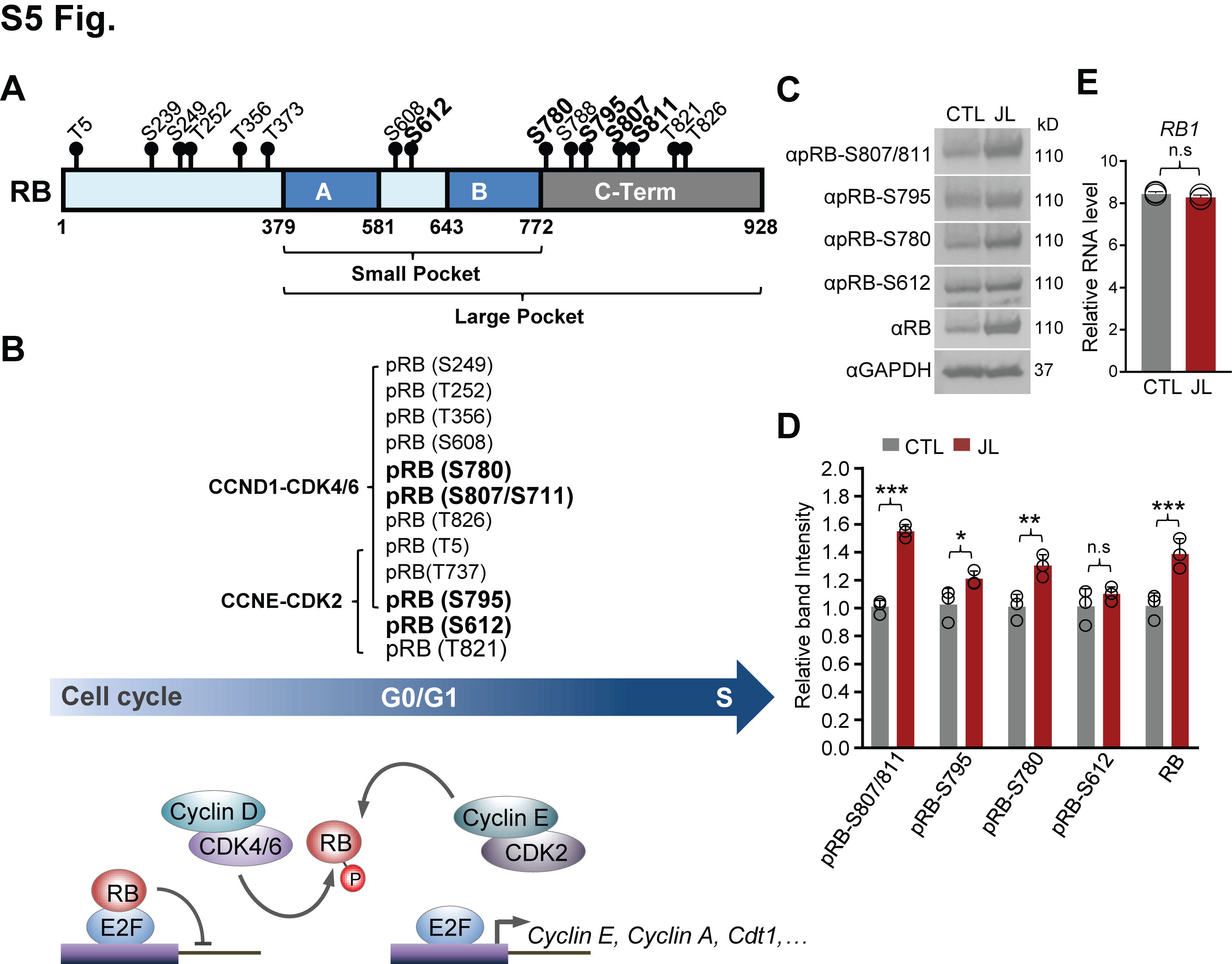

Supplement: S5 Fig — (A) Schematic representation of CDK phosphorylation sites in human RB. Position of the consensus Cdk phosphorylation sites in relation to the RB protein is indicated. The A and B domains of the small pocket and large pocket and the carboxyl terminus are indicated. (B) Schematic representation of the cyclin D1-CDK4/6 and/or cyclin E-CDK2 phosphorylation sites in RB required for G0/G1/S phase transition. Complexes involved in this transition are also indicated. Phosphorylation sites (pRB-S807/811, pRB-S795, pRB-S780, and pRB-S612) assayed in subsequent western blot analysis of RB phosphorylation status are highlighted in bold. (C) Western blot (WB) analysis of total RB or phospho-RB proteins (pRB-S807/811, pRB-S795, pRB-S780, pRB-S612), with specific antibodies as indicated in control (CTL) and jet lag (JL) cells 24 hours after the final dex stimulation, as per the experimental schedule depicted in Fig 1A. Anti-GAPDH (αGAPDH) was used for loading control. (D) Statistical analysis of WB data in (C) showing the total or phosphorylated RB proteins at multiple sites as indicated (*p < 0.05, **p < 0.01, ***p < 0.001 by two-way ANOVA and Bonferroni multiple comparisons test). Data normalized are represented as mean ± SD from n = 3 independent experiments. CTL (grey bar); JL (brown bar). (E) Comparison of RB1 expression profiles in CTL (grey bar) and JL (brown bar) cells from RNA sequencing data. n.s., p > 0.05. Data normalized are shown with means ± SD; n = 3. Underlying data are provided in S3 Data. CCD, chronic circadian desynchrony; CDK, cyclin dependent kinase; CTL, control; dex, dexamethasone; JL, jet lag; n.s., not significant; P, phosphorylation; pRB, phospho-RB protein; RB, retinoblastoma; WB, western blot. (TIF) [file pbio.3000228.s005.tif]

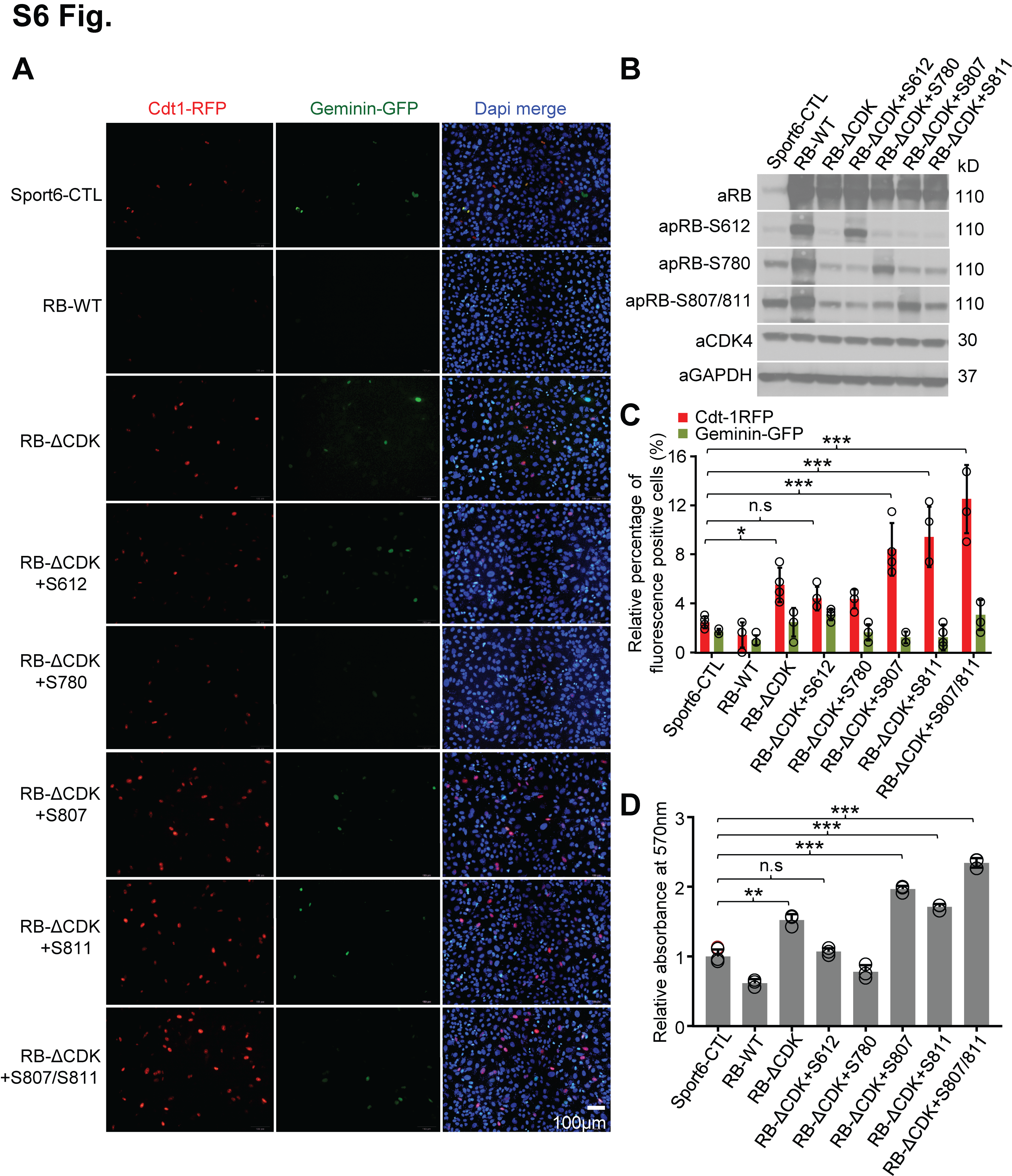

Supplement: S6 Fig — (A) Forty-eight hours after transfection of the control (Sport6-CTL), the intact RB (RB-WT), ΔCdk RB (RB-ΔCDK), and RB-single-Cdk site constructs in U2OS cells, the cells were transduced with baculovirus-expressing FUCCI cell cycle sensors (Cdt1-RFP for G1/S, Geminin-GFP for G2/M) for 48 hours and were fixed for microscopic analysis. Numbering indicates single Cdk site location on RB. Representative images were captured by fluorescence imaging microscopy using specific filter sets for FITC (grey green; Geminin-GFP), TRITC (red; Cdt-RFP), and DAPI (blue; nuclei). (B) Immunoblot of the intact RB (RB-WT), ΔCdk RB (RB-ΔCDK), and RB-single-Cdk site proteins expressed in U2OS cells using the antibodies indicated. GAPDH (αGAPDH) is loading control. (C) Quantification of the fraction of FUCCI cell cycle indicator–positive cells in the image data shown in (A). Proportion of Geminin-GFP (grey green bar)–or Cdt1-RFP (red bar)–positive cells from the total number of Dapi-stained nuclei (>250) in each of the image panels indicated were averaged from four optical fields scanned with a 20× objective. *p < 0.05, ***p < 0.001 (two-way ANOVA and Tukey multiple comparison test). Data were normalized and represented as mean ± SD; n = 4. The result is representative of three independent experiments. (D) Forty-eight hours after transfection of control (Sport6-CTL), intact RB (RB-WT), ΔCdk RB (RB-ΔCDK), and RB-single-Cdk site constructs as indicated in U2OS cells, the cells were incubated in the changed media for 48 hours followed by MTT assay for evaluation of cell proliferation. **p < 0.005, ***p < 0.0001, two-tailed t test. Data were normalized and represented as mean ± SD; n = 3. The result is representative of three independent experiments. Underlying data are provided in S3 Data. Cdk, cyclin-dependent kinase; Cdt1-RFP, chromatin licensing and DNA replication factor 1 tagged with red fluorescent protein; FITC, fluorescein isothiocyanate; FUCCI, fluorescence ubiquitination-based cell- [file pbio.3000228.s006.tif]

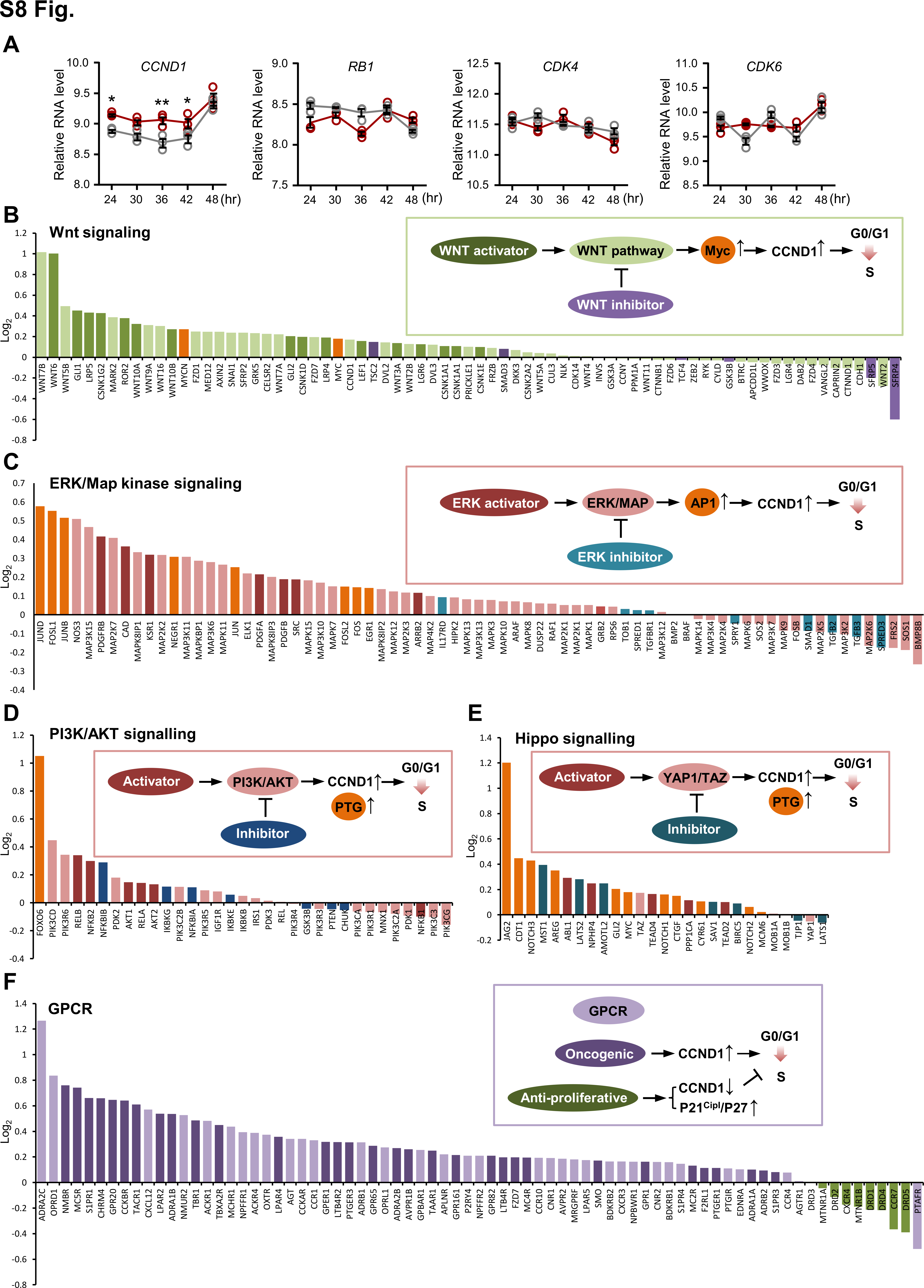

Supplement: S8 Fig — (A) Comparison of cyclin D1, RB1, CDK4, and CDK6 mRNA expression profiles from RNA sequencing data in control (CTL; grey circle) and jet lag (JL; brown circle) cells collected every 6 hours, as indicated, for 24 hours following the chronic desynchronization schedule depicted in Fig 1A. *p < 0.05, **p < 0.005; two-way ANOVA with Bonferroni multiple comparisons test. Data are shown with the means ± SEM; n = 3 in all time points. (B-F) Log2 fold-change values for gene expression of key activators, mediators, or repressors of (B) Wnt, (C) ERK/MAPK, (D) PI3K/AKT, (E) Hippo, (F) GPCR signaling pathways. Fold-change values calculated using RNA sequencing data from CTL and JL cells. Schematics for cellular pathways known to regulate cyclin D1 (CCND1) or pathway target gene (PTG) expression for G0/G1/S phase cell cycle progression are included as panel insets. Colors of bars in fold-change graphs correspond to associated components in the pathway diagrams. See S10, S11, S12, S13 and S14 Tables. CCND1, cyclin D1; CTL, control; dex, dexamethasone; ERK/MAPK, extracellular signal-regulated kinase/mitogen activated protein kinase; GPCR, G protein-coupled receptor; Hippo, hippo signaling pathway; JL, jet lag; PI3K/AKT, phosphatidylinositol 3-kinase/alpha serine/threonine-protein kinase; PTG, pathway target gene; RB, retinoblastoma; RB-WT, wild-type RB; Wnt, wingless/Integrated. (TIF) [file pbio.3000228.s008.tif]

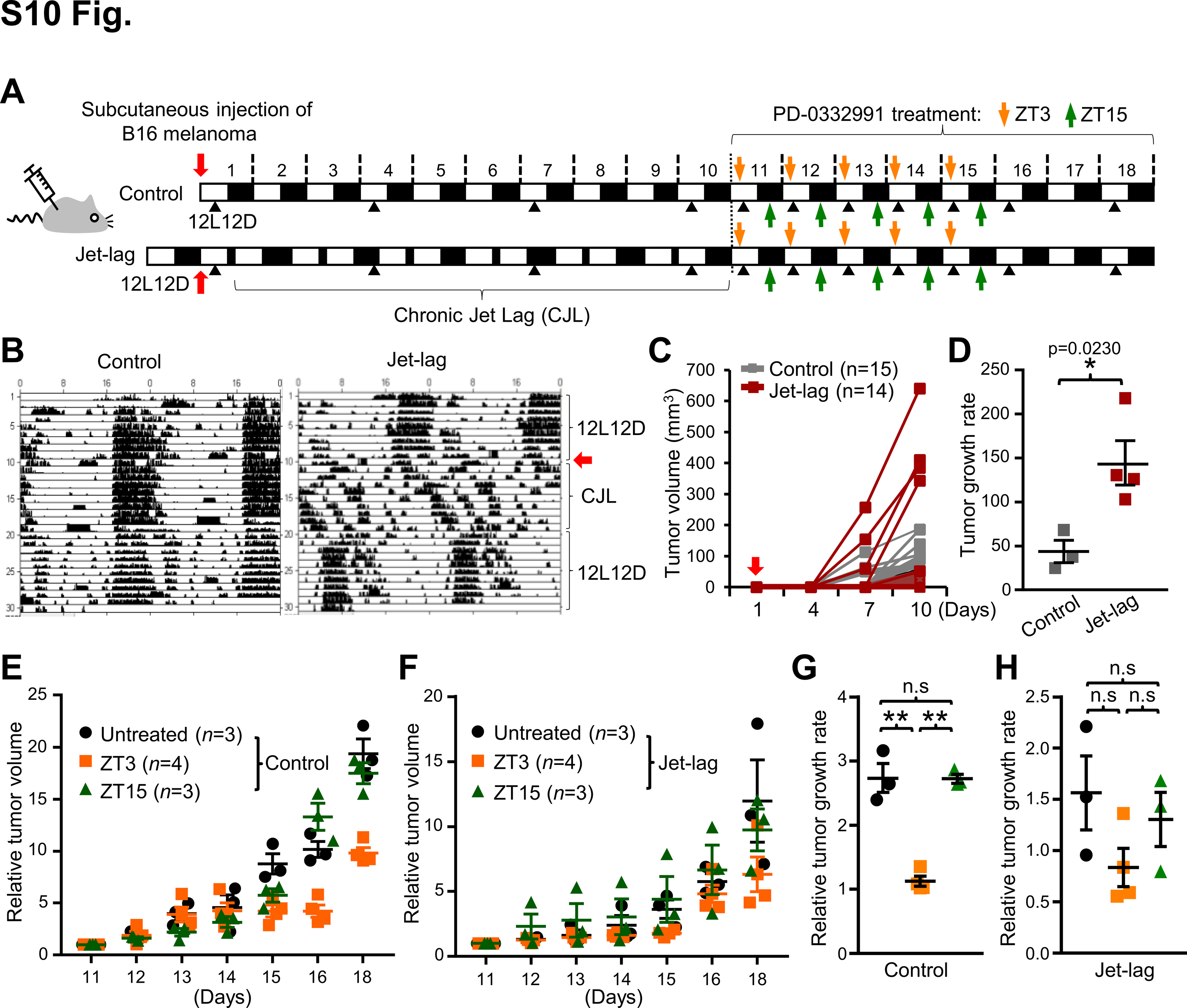

Supplement: S10 Fig — (A) The experimental schedule for chronic jet lag and palbociclib (PD-0332991) drug treatment. The red arrow indicates subcutaneous injection of B16 mouse melanoma cells (1 × 10^6^). Black arrowheads denote times of tumor measurement. Orange and green arrows indicate oral drug administration of mice at ZT3 and ZT15. Treatment started on day 11 after tumor inoculation. (B) Representative activity records of running wheel activity in Control and Jet-lag mice. The red arrow indicates B16 melanoma injection. (C) Plots depicting tumor growth in Control (grey square, n = 15) and Jet-lag (brown square, n = 14) mice during chronic jet lag. Red arrow indicates B16 melanoma injection. (D) Quantification of melanoma tumor growth rate calculated from linear regression by fitting a linear equation to observed data in Control (n = 3; grey square) and Jet-lag (n = 4; brown square) mice of (C). *p < 0.05, two-tailed and paired Student t test. Data normalized were shown with mean ± SEM. (E and F) Time-dependent effects of palbociclib on melanoma tumor growth in Control (E) or Jet-lag (F) mice. Tumor growth changed as a function of palbociclib administration time; untreated (black circle), treated at ZT3 (orange square), treated at ZT15 (green triangle). n indicates number of mice analyzed. Data normalized were shown with mean ± SEM; n = 3–4 per group. The result is representative of two independent experiments. (G and H) Quantification of melanoma tumor growth rate calculated from linear regression by fitting a linear equation to observed data in Control (G) or Jet-lag mice (H) under the different drug treatment conditions; untreated (black circle), treated at ZT3 (orange square), or treated at ZT15 (green triangle). n = 3–4 mice were analyzed per group. **p < 0.001, one-way ANOVA and Tukey multiple comparison test. Data were shown with mean ± SEM. Underlying data are provided in S3 Data. n.s., not significant; ZT, zeitgeber time. (TIF) [file pbio.3000228.s010.tif]
